# Supplementary material for: Effect of simulated handball match-induced fatigue on isokinetic hamstring-to-quadriceps ratio and evertor-to-invertor ratio in professional players
Source: PLoS One. 2025 May 19;20(5):e0323978. doi: 10.1371/journal.pone.0323978 (PMC12088022; doi:10.1371/journal.pone.0323978)
Supplement: Attachment — Submitted filename: PONE-D-25-07828_reviewer_reviewer comments.pdf [file pone.0323978.s001.pdf]

PLOS ONE

Effect of simulated handball match-induced fatigue on isokinetic hamstring-to-quadriceps ratio and invertor-to-evertor ratio in professional players

--Manuscript Draft--

**Manuscript Number:**	PONE-D-25-07828
**Article Type:**	Research Article
**Full Title:**	Effect of simulated handball match-induced fatigue on isokinetic hamstring-to-quadriceps ratio and invertor-to-evertor ratio in professional players
**Short Title:**	Effect of simulated handball match-induced fatigue on lower limb peak torques
**Corresponding Author:**	Hamed Esmaeili, Ph.D<br>University of Isfahan<br>Isfahan, Isfahan IRAN, ISLAMIC REPUBLIC OF
**Keywords:**	Isokinetic; Knee; Ankle; fatigue; Handball; hamstring-to-quadriceps ratio; Torque; Injury risk
**Abstract:**	Knee and ankle injuries are of the most common injuries in the handball players. Many of these injuries occur towards the end of the game when fatigue sets. The underlying mechanisms of this phenomenon are not well understood. This study investigated the effect of a handball match-simulation protocol on the hamstring-to-quadriceps (H:Q) and evertor-to-invertor (E:I) peak torque ratios. Thirty professional male handball players from Iranian super league teams participated in this study. Isokinetic concentric peak torque of the hamstrings, quadriceps, ankle invertor, and ankle evertors were measured at angular velocities of 60, 120, and 180 deg/s) before and after the simulated handball match-induced fatigue protocol using a Biodex Isokinetic Dynamometer. Peak torque ratio of H:Q and E:I were calculated and compared between pre- and post-fatigue conditions. Fatigue significantly reduced H:Q peak torque ratio at 60 (p=0.049), 120 (p=0.002) and 180 (p=0.014) deg/s, as well as E:I peak torque ratio at 60 (p=0.010), 120 (p=0.003) and 180 (p=0.003) deg/s. These changes could contribute to an increased risk of anterior cruciate ligament (ACL) tears and lateral ankle sprains. Given the greater reduction in the H:Q peak torque ratio in hamstring muscles, targeted strengthening and improving the endurance of these muscles is recommended for professional handball players to mitigate fatigue effects.
**Order of Authors:**	Ali Shirani Dastjerdi
	Hamed Esmaeili, Ph.D
	Morteza Sadeghi
	Behzad Bashiri
**Additional Information:**	
**Question**	**Response**
**Financial Disclosure**<br><br>Enter a financial disclosure statement that describes the sources of funding for the work included in this submission. Review the submission guidelines for detailed requirements. View published research articles from *PLOS ONE* for specific examples.<br><br><br>This statement is required for submission and **will appear in the published article** if	The author(s) received no specific funding for this work.

Powered by Editorial Manager® and ProduXion Manager® from Aries Systems Corporation

the submission is accepted. Please make sure it is accurate.<br><br>**Funded studies**<br>Enter a statement with the following details:<br>• Initials of the authors who received each award<br>• Grant numbers awarded to each author<br>• The full name of each funder<br>• URL of each funder website<br>• Did the sponsors or funders play any role in the study design, data collection and analysis, decision to publish, or preparation of the manuscript?<br><br>Did you receive funding for this work?	
**Competing Interests**<br><br>Use the instructions below to enter a competing interest statement for this submission. On behalf of all authors, disclose any competing interests that could be perceived to bias this work—acknowledging all financial support and any other relevant financial or non-financial competing interests.<br><br>This statement is required for submission and **will appear in the published article** if the submission is accepted. Please make sure it is accurate and that any funding sources listed in your Funding Information later in the submission form are also declared in your Financial Disclosure statement.<br><br>View published research articles from *PLOS ONE* for specific examples.	The authors have declared that no competing interests exist.

**NO authors have competing interests**<br><br>Enter: *The authors have declared that no competing interests exist.*<br><br>**Authors with competing interests**<br><br>Enter competing interest details beginning with this statement:<br><br>*I have read the journal's policy and the authors of this manuscript have the following competing interests: [insert competing interests here]*<br><br>* typeset	
**Ethics Statement**<br><br>Enter an ethics statement for this submission. This statement is required if the study involved:<br><br>• Human participants<br>• Human specimens or tissue<br>• Vertebrate animals or cephalopods<br>• Vertebrate embryos or tissues<br>• Field research<br><br>Write "N/A" if the submission does not require an ethics statement.<br><br>General guidance is provided below. Consult the submission guidelines for detailed instructions. **Make sure that all information entered here is included in the Methods section of the manuscript.**	Human Subject Research:<br>- institutional review board: Research Ethics Committee, University of Isfahan<br>- Approval Note from the Research Ethics Committee: the project was found to be in accordance with the ethical principles and the national norms and standards for conducting Medical Research in Iran.<br>Notice:<br>1. Although the proposal has been approved by the Biomedical Research Ethics Committee,<br>meeting the professional and legal requirements is the sole responsibility of the PI and other project collaborators.<br>2. This certificate is reliant on the proposal/documents received by this committee on 2022-08-<br>06. The committee must be notified by the PI as soon as the proposal/documents are modified.<br><br>-form of consent obtained: Written consent

Powered by Editorial Manager® and ProduXion Manager® from Aries Systems Corporation

**Format for specific study types**<br><br>**Human Subject Research (involving human participants and/or tissue)**<br>• Give the name of the institutional review board or ethics committee that approved the study<br>• Include the approval number and/or a statement indicating approval of this research<br>• Indicate the form of consent obtained (written/oral) or the reason that consent was not obtained (e.g. the data were analyzed anonymously)<br><br>**Animal Research (involving vertebrate animals, embryos or tissues)**<br>• Provide the name of the Institutional Animal Care and Use Committee (IACUC) or other relevant ethics board that reviewed the study protocol, and indicate whether they approved this research or granted a formal waiver of ethical approval<br>• Include an approval number if one was obtained<br>• If the study involved *non-human primates*, add *additional details* about animal welfare and steps taken to ameliorate suffering<br>• If anesthesia, euthanasia, or any kind of animal sacrifice is part of the study, include briefly which substances and/or methods were applied<br><br>**Field Research**<br><br>Include the following details if this study involves the collection of plant, animal, or other materials from a natural setting:<br>• Field permit number<br>• Name of the institution or relevant body that granted permission	
**Data Availability**<br><br>Authors are required to make all data underlying the findings described fully available, without restriction, and from the time of publication. PLOS allows rare exceptions to address legal and ethical concerns. See the PLOS Data Policy and FAQ for detailed information.	Yes - all data are fully available without restriction

A Data Availability Statement describing where the data can be found is required at submission. Your answers to this question constitute the Data Availability Statement and **will be published in the article**, if accepted.<br><br>**Important:** Stating 'data available on request from the author' is not sufficient. If your data are only available upon request, select 'No' for the first question and explain your exceptional situation in the text box.<br><br>Do the authors confirm that all data underlying the findings described in their manuscript are fully available without restriction?	
**Describe where the data may be found in full sentences. If you are copying our sample text, replace any instances of XXX with the appropriate details.**<br><br>• If the data are **held or will be held in a public repository**, include URLs, accession numbers or DOIs. If this information will only be available after acceptance, indicate this by ticking the box below. For example: *All XXX files are available from the XXX database (accession number(s) XXX, XXX.).*<br>• If the data are all contained **within the manuscript and/or Supporting Information files**, enter the following: *All relevant data are within the manuscript and its Supporting Information files.*<br>• If neither of these applies but you are able to provide **details of access elsewhere**, with or without limitations, please do so. For example:<br><br>*Data cannot be shared publicly because of [XXX]. Data are available from the XXX Institutional Data Access / Ethics Committee (contact via XXX) for researchers who meet the criteria for access to confidential data.*<br><br>*The data underlying the results presented in the study are available from (include the name of the third party*	NA

and contact information or URL).<br>• This text is appropriate if the data are owned by a third party and authors do not have permission to share the data.<br><br>* typeset	
Additional data availability information:	Tick here if your circumstances are not covered by the questions above and you need the journal's help to make your data available.; Tick here if the URLs/accession numbers/DOIs will be available only after acceptance of the manuscript for publication so that we can ensure their inclusion before publication.

Powered by Editorial Manager® and ProduXion Manager® from Aries Systems Corporation

Manuscript
Click here to access/download;Manuscript;Fin Handball-bb000.docx

Effect of simulated handball match-induced fatigue on isokinetic hamstring-to-quadriceps ratio and invertor-to-evertor ratio in professional players

Ali Shirani Dastjerdi[1], Hamed Esmaeili[1*], Morteza Sadeghi[1], Behzad Bashiri[2]

[1] Department of Sport Injuries and Corrective Exercises, Faculty of Sport Sciences, University of Isfahan, Isfahan, Iran

[2] Department of Biomedical Engineering, Faculty of Engineering, International University of Science and Technology in Kuwait, Ardiya, Kuwait

Short Title: Effect of simulated handball match-induced fatigue on lower limb peak torques

* Corresponding author: Hamed Esmaeili

Email: H.esmaeili@spr.ui.ac.ir

Abstract

Knee and ankle injuries are of the most common injuries in the handball players. Many of these injuries occur towards the end of the game when fatigue sets. The underlying mechanisms of this phenomenon are not well understood. This study investigated the effect of a handball match-simulation protocol on the hamstring-to-quadriceps (H:Q) and evertor-to-invertor (E:I) peak torque ratios. Thirty professional male handball players from Iranian super league teams participated in this study. Isokinetic concentric peak torque of the hamstrings, quadriceps, ankle invertor, and ankle evertors were measured at angular velocities of 60, 120, and 180 deg/s) before and after the simulated handball match-induced fatigue protocol using a Biodex Isokinetic Dynamometer. Peak torque ratio of H:Q and E:I were calculated and compared between pre- and post-fatigue conditions. Fatigue significantly reduced H:Q peak torque ratio at 60 ($p=0.049$), 120 ($p=0.002$) and 180 ($p=0.014$) deg/s, as well as E:I peak torque ratio at 60 ($p=0.010$), 120 ($p=0.003$) and 180 ($p=0.003$) deg/s. These changes could contribute to an increased risk of anterior cruciate ligament (ACL) tears and lateral ankle sprains. Given the greater reduction in the H:Q peak torque ratio in hamstring muscles, targeted strengthening and improving the endurance of these muscles is recommended for professional handball players to mitigate fatigue effects.

Keywords: Isokinetic, Knee, Ankle, Fatigue, Handball, hamstring-to-quadriceps ratio, Torque, Injury risk

Introduction

Handball is a complex, highly demanding intermittent sport that involves multiple high-intensity runs (1), frequent body contact, and other high-intensity actions to overcome opponents. A handball match involves a large number of repeated accelerations, sprints, jumps, blocking, pushing and rapid changes in moving directions (2). Sudden changes of direction, decelerations and accelerations, vertical and horizontal jumps, throws, thrusts, pulls, and powerful, fast-paced clashes are among the movements of handball sport that have made it a sport with high risk of injury (3).

These physical demands are expected to lead to fatigue, which could be at least partially responsible for the reduction in the quantity and quality of high intensity actions towards the end of a match compared to the beginning (4). It has been shown that players who play 70 percent of the total match time experience changes such as reduction in high-intensity running, reduction in heart rate, reduction in technical and impaired physical performance, resulting from fatigue (5). This condition could increase demand on musculoskeletal system and leaves players at high risk of injury. Notably, the highest number of injuries during six periods of international competitions occurred between minutes 16 to 30 of the first half and 46 to 60 of the second half (6).

Given these findings, it can be suggested that physical fatigue deteriorates neuromuscular and sensorimotor control, which may elevate the risk of injury. Handball players report a higher prevalence of lower limb injuries than injuries to the upper limbs, neck, or head (6,7). For instance, approximately 30% of injuries occur in the knee, and 25% in the ankle (7).

The hamstring-to-quadriceps (H:Q) peak torque ratio in the knee joint and evertor to invertor (E:I) peak torque ratio in the ankle are two risk factors for determining muscle balance or imbalance in the knee and ankle (8–11). Deterioration in these ratios can increase the risk of injury. A decreased H:Q ratio implies that the quadriceps are disproportionately stronger than the hamstrings, which stresses the anterior cruciate ligament (ACL) and may lead to injuries (12). A decrease in the E:I peak torque ratio is linked to a higher risk of lateral ankle sprains (13).

Isokinetic muscle peak torque is a critical metric in assessing injury risk, particularly in sports and rehabilitation settings. Isokinetic testing can identify neuromuscular deficits, such as reduced torque production or delayed muscle activation, which are often linked to injury risk (14,15). For example, a decline in hamstring peak torque has been associated with an increased risk of hamstring strains in soccer players (15).

Given the high incidence of lower limb injuries among handball players, particularly when players are fatigued during matches, it is crucial to investigate the effects of match-induced fatigue on H:Q and E:I peak torque ratios in handball. The aim of this study is to evaluate the effect of simulated handball match-induced fatigue on the isokinetic hamstring-to-quadriceps and invertor-to-evertorratios ratios in professional handball players.

Materials and Methods

Participants

Thirty professional male handball players from Iranian super league teams (age: 27 ± 3.1 years; height: 186.9 ± 7.3 cm; mass: 86.8 ± 6.7 kg) participated in this study. Participant recruitment for this study began on Augost 15, 2023, and concluded on November 30, 2023. All participants were active team members and played in

each match. Volunteers who reported injuries in the lower limbs in the last 6 months wer excluded. All participants provided written informed consent. This study was approved by the University of Isfahan Ethics Committee and conducted in accordance with the Declaration of Helsinki.

Testing preparation

Initially, participants were briefed on the testing procedure. After a preliminary warm up, they sat on the dynamometer chair (Biodex Multi-joint System 3, Biodex Medical Systems, USA) which was adjusted so they had free and full movement in knee and ankle joints. Participants were fixed with two horizontal and vertical belts, and the test limb was fixed to the arm of the device. The seat of the device was adjusted to an angle of 100 degrees. The arm of the device was adjusted to a tilit angle of 0 degree°. The movement axis of the knee was aligned parallel to the movement axis of the device. For the ankle test, the participant's ankle was positioned on the footplate and the foot was secured using two Velcro straps.

For each test session, the range of motion of ankle and knee joints was determined first (16). Concentric isokinetic peak torque for quadriceps, hamstring, ankle invertors, and ankle evertors was measured at three different angular velocities of 60, 120 and 180 deg/s by a Biodex Multi-joint System 3 dynamometer (Biodex Medical Systems, USA) on the landing leg in a random order. The landing leg was defined as the leg that a player uses to land after jumping (17,18). Each angular velocity was measured with one set of six repetitions. In each test, participants were instructed to perform the movements with maximum effort. Verbal encouragement was provided throuought the test. Peak torques were assessed at baseline and immediately after the handball-specific fatigue protocol to examine its effects on the outcomes.

Fatiguing protocol

A previously used fatigue protocol was utilized to simulate match-induced fatigue (19) which included special movements during a handball match, such as sprinting, jumping, landing, sidestep cutting, and lateral and backward shifting (Fig 1, caption for more details). The protocol was divided into gradually increasing rounds, requiring participants to complete each round as quickly as possible. The number of rounds corresponded to the number of laps in the circuit (e.g., round I: 1 lap; round II: 2 laps; and so on). For each round, time of effort, heart rate using Polar F4 Blue ICE, and the rate of perceived exertion (RPE) using the 15-point Borg scale with a a range of 6-20 were recorded (20). In this Borg Scale, a score of six indicates no fatigue, while scores between 17 and 20 indicate the inability to continue the exercises. During the trials, if participants reported a score of 17 or higher at the end of a round, the fatigue protocol ended. If they reported a lower score, they performed another round until they reached a score of 17.

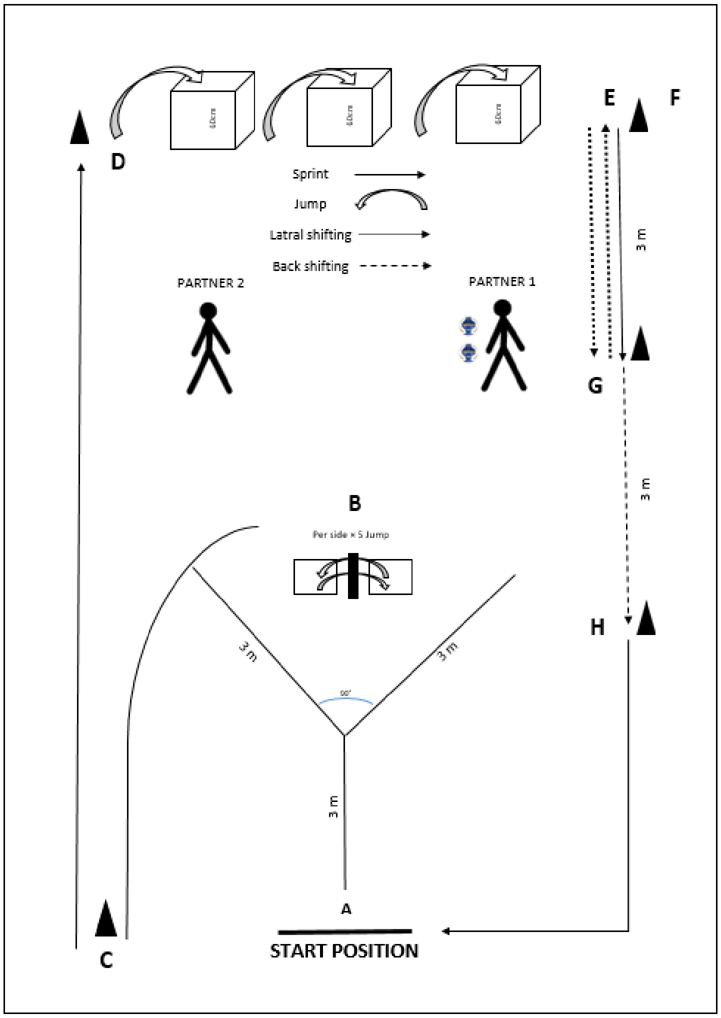

Fig 1. Illustration of the fatigue protocol based on specific movements performed by handball athletes. The athletes should perform the following actions in all of the stations as fast as possible: (A) Acceleration and sidestep cutting maneuver for the right and run until the end of the 3-m distance; receive a pass (at the end of the 3-m length) from a partner positioned 2.5 m away (Partner 1); backshift until the 90°; run 3 m for the left side, and perform an accurate throw to a partner positioned 2.5 m away (Partner 2); this task was performed twice per lap; (B) Ten double-leg lateral jumping/landing over a barrier (20-cm high), performed as fast as possible; (C) Sprint; (D) Double-leg plyometric jumps, performed as fast as possible; (E) Lateral shifting; (F) Sprint; (G) Back Shifting; and (H) Sprint with a 90° change of direction. The protocol was divided into rounds, where the round number represents the number of laps in the circuit (e.g., round I: 1 lap, round II: 2 laps, round III: 3 laps, and so on) until the participant is unable to continue performing the protocol.

Data analysis

Peak knee flexion, knee extension, ankle inversion and ankle eversion torque were recorded for each repetition. Between each set of the test, participants rested for 2-min. To calculate the H:Q ratio, peak flexion torque was divided by the peak extensor torque [15 Bilateral]. To calculate the E:I ratio, peak eversion torque was divided by the peak inversion torque (13). Peak torques per body weight, along with the peak torque ratios of H:Q and E:I, were used in subsequent analyses.

Statistical Analysis

Shapiro-Wilk test was used to ensure normality of data distribution. To investigate the effect of fatigue on the outcomes, a 2 (condition: pre- and post-fatigue) × 3 (angular velocity: 60, 120, and 180 deg/s) repeated measures ANOVA was used. Holm-Bonferroni adjustment was used for multiple comparisons. Effect size (ES), reported using partial eta squared, η_p^2, was classified as small (η_p^2 = 0.01), medium (η_p^2 = 0.06), and large (η_p^2 = 0.14) (21). All statistical analyses were performed at a significance level of $p < 0.05$ using SPSS software, version 18.0 (SPSS Inc, Chicago IL, USA).

Results

Effect of fatigue on peak torques

The results showed that the main effect of fatigue ($F_{(1,29)} = 36.14$, $p < 0.01$, ES = 0.56) and the main effect of angular velocity ($F_{(2,58)} = 87.58$, $p < 0.01$, ES = 0.751) were significant, but the interaction effect was not significant ($F_{(2,58)} = 2.20$, p = 0.120, ES = 0.070) on hamstring peak torque ratio. Multiple comparisons showed that fatigue decreased hamstring peak torque in all angular velocities (Table 1).

For quadriceps peak torque, the results showed significant main effects for fatigue ($F_{(1,29)} = 7.18$, p = 0.012, ES = 0.198) and angular velocity ($F_{(2,58)} = 296.68$, p = 0.001, ES = 0.911), and significant fatigue × angular velocity interaction effect ($F_{(2,58)} = 4.20$, p = 0.020, ES = 0.127). This indicates that fatigue affects quadriceps peak torque differently at various angular velocities. Post-hoc tests revealed that fatigue was associated with a decrease in quadriceps peak torque at 60 deg/s and 180 deg/s, but not at 120 deg/s (Table 1).

Table 1. **Comparison of hamstring and quadriceps peak torque ratio in the pre- and post-fatigue at different angular velocities. Data are presented in Mean ± SD.**

	Angular Velocity	Pre	Post	Mean Difference	Standard Error	t	p_{holm}
**Hamstring**	60 deg/s	1.83 ± .36	1.56 ± .25	.27333	.0649	4.214	**.001**
	120 deg/s	1.55 ± .24	1.43 ± .26	.12247	.0360	3.398	**.006**
	180 deg/s	1.32 ± .23	1.18 ± .23	.13907	.0198	7.031	**.001**
**Quadriceps**	60 deg/s	2.84 ± .35	2.65 ± .43	.1827	.0649	2.81	**.026**
	120 deg/s	2.33 ± .34	2.24 ± .31	.0852	.0564	1.51	.141
	180 deg/s	1.68 ± .25	1.60 ± .27	.9749	.0668	2.19	**.041**

Bold values indicate significant difference in $p \leq 0.05$.

Regarding the invertor peak torque ratio, the results of ANOVA showed significant main effect of fatigue ($F_{(1,29)} = 12.97$, p = 0.001, ES = 0.309) and angular velocity ($F_{(2,58)} = 71.46$, p = 0.001, ES = 0.711), but no significant fatigue × angular velocity interaction ($F_{(2,58)} = 2.11$, p = 0.130, ES = 0.068). Multiple comparisons showed that, after fatigue, invertor peak torque decreased only in 60 deg/s (Table 2).

Repeated measures analysis of variance showed significant main effects of fatigue ($F_{(1,29)} = 44.01$, p = 0.001, ES = 0.603) and angular velocity ($F_{(2,58)} = 99.48$, p = 0.001, ES = 0.774), and significant fatigue by angular velocity interaction ($F_{(2,58)} = 3.35$, p = 0.042, ES = 0.104) on evertor peak torque. The handball-specific fatigue protocol was associated with decrease in evertor peak torque in all three angular velocities (Table 2).

Table 2. Comparison of invertor and evertor peak torque ratio in the pre- and post-fatigue at different angular velocities. Data are presented in Mean ± SD.

		Pre	Post	Mean Difference	Standard Error	t	p_{holm}
**Invertor**	60 deg/s	.44 ± .10	.39 ± .10	.0469	.01233	3.80	**.003**
	120 deg/s	.37 ± .08	.35 ± .09	.0240	.01194	2.01	.161
	180 deg/s	.31 ± .09	.30 ± .06	.0165	.01179	1.40	.345
**Evertor**	60 deg/s	.42 ± .12	.33 ± .08	.08779	.01389	6.319	**.001**
	120 deg/s	.35 ± .09	.27 ± .06	.07345	.01292	5.685	**.001**
	180 deg/s	.27 ± .08	.21 ± .05	.05667	.01202	4.714	**.001**

Bold values indicate significant difference in $p \leq 0.05$.

Effect of fatigue on peak torque ratios

ANOVA test showed significant main effects of fatigue ($F_{(1,29)} = 18.143$, $p = 0.001$, ES = 0.385) and angular velocity ($F_{(2,58)} = 60.019$, $p = 0.001$, ES = 0.674), but no significant fatigue × angular velocity interaction effect ($F_{(2,58)} = 0.526$, $p = 0.594$, ES = 0.018) on H:Q peak torque ratio. Fig 2 illustrates H:Q peak torque ratio in the pre- and post-fatigue in different angular velocities. As shown, fatigue decreased H:Q ratio in 60 ($t_{(1,29)} = 2.662$, $p = 0.049$), 120 ($t_{(1,29)} = 4.202$, $p = 0.002$) and 180 ($t_{(1,29)} = 3.327$, $p = 0.014$) deg/s.

ANOVA test showed significant main effects for fatigue ($F_{(1,29)} = 17.39$, $p = 0.001$, ES = 0.375) and angular velocity ($F_{(2,58)} = 29.90$, $p = 0.001$, ES = 0.508), but no significant fatigue by angular velocity interaction effect ($F_{(2,58)} = 1.63$, $p = 0.206$, ES = 0.053) on E:I. As shown in Fig 3, fatigue decreased E:I in 60 ($t_{(1,29)} = 3.469$, $p = 0.010$), 120 ($t_{(1,29)} = 4.164$, $p = 0.003$) and 180 ($t_{(1,29)} = 4.198$, $p = 0.003$) deg/s.

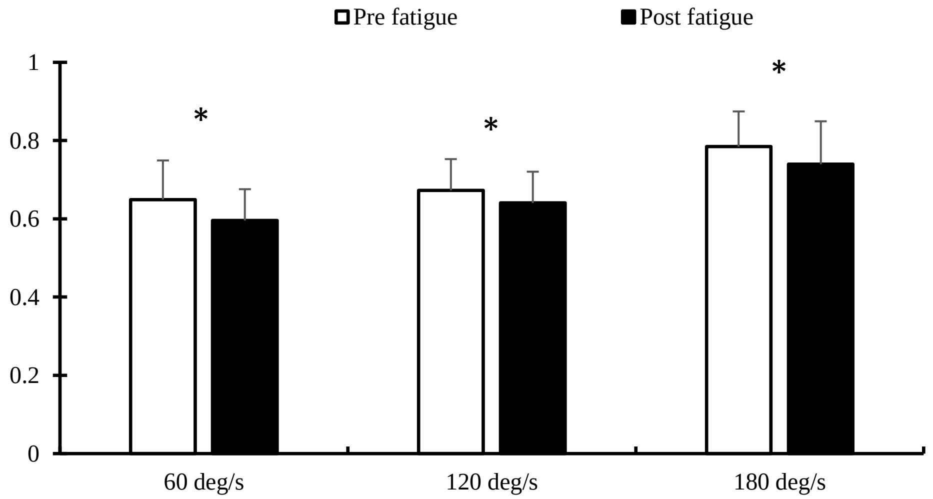

Fig 2. Comparison of H:Q peak torque ratio at different angular velocities in pre- and post-fatigue. (*) stands for significant difference in $p \leq 0.05$.

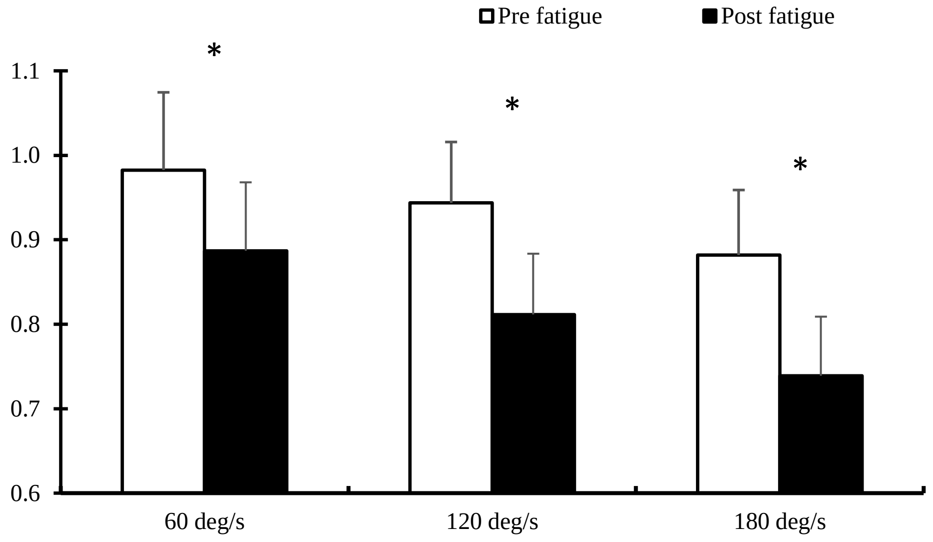

Fig 3. Comparison of E:I peak torque ratio in different angular velocities in pre-and-post fatigue. (*) stands for significant difference in $p \leq 0.05$.

Discussion

The aim of the current study was to investigate the effect of simulated handball match-induced fatigue on H:Q and E:I peak torque ratios in Iranian handball super league players. Our results showed that fatigue decreased peak hamstring and quadriceps muscles torque and H:Q peak torque ratio. Also, the results showed that fatigue was associated with a decrease in peak invertor and evertor muscle torque and decrease in E:I peak torque ratio. In line with our results, Pinto *et al*. and Sangnier and Tourny-Chollet showed decrease in knee flexor and extensors torque and hamstring to quadriceps peak torque ratio decrease (11,22). Massamba *et al*. showed that after training-induced fatigue, the maximum voluntary isometric force of quadriceps and hamstring muscles decreased (23). Observed decreases in hamstring and quadriceps muscle peak torque indicate effect of a simulated handball match-induced fatigue on knee flexor and extensor muscles strength and force output. While it has been well known that fatigue affects peak force output, training modalities seem to have different effects on force output reduction (24–28). After

completion of different fatigue protocols, similar reductions in force output are observed in knee flexor and extensor muscles concentric or isometric measurements (23,25). Decrease in strength and torque output could be due to general fatigue or fatigue in local muscles. To better explain mechanisms leading to a decrease in force output of muscles, changes in muscle activation can be pointed out; it has been shown that general fatigue can result from disturbances in CA^{2+} during contraction (29). Moreover, local fatigue can be induced by disturbances in stretch-shortening cycle, changes in cross-bridge characteristics, or metabolic changes (30).

Our results showed that knee flexor muscles are influenced by handball match induced fatigue as well as knee extensors. Changes in fatigued condition are repetitively reported for knee extensor muscles (31,32). However, our results are inconsistent with those of Marshal *et al.* who showed soccer simulation match-induced fatigue has no effect on hamstring muscles peak torque (31). This inconsistency could be attributed to differences in the population and fatigue protocol. Marshal *et al.* studied soccer players and a soccer simulation match, whereas the current study has included professional handball players and used a handball match-simulating protocol which engaged both hamstring and quadriceps muscles.

Our results showed that H:Q peak torque ratio decreased after fatigue protocol. The reduction in this ratio was associated with a decrease in hamstring and quadriceps muscles peak torques. Since this ratio is calculated by dividing hamstring peak torque by quadriceps peak torque, it seems that hamstring muscles are more affected by fatigue. Pinto *et al.* showed that after a fatigue protocol, hamstring muscles torque decreases to a greater extent than that of quadriceps (22), which is in line with the results of our study. Hamstring muscles besides their complex muscular architecture and multi-joint function, comprise a significant number of type II fibers and are less

utilized in most daily activities, making them more susceptible to muscle damage and fatigue (33,34). Although the mechanism of hamstring and ACL injuries is still a subject of debate, it is well-established that neuromuscular fatigue is an important risk factor. For example, changes in hamstring muscle coordination, alterations in hamstring length during late swing phase of running cycle, and altered ACL-hamstring reflex are associated with muscle stiffness regulation and knee stability after a fatigue protocol. Overall, these findings also indicate that knee flexors should be well-trained to enhance their force production capacity and endurance, reducing susceptibility to hamstring and ACL injuries. Additionally, a reduction in the H:Q peak torque ratio increases the risk of ACL injury (11).

The results of the present study demonstrated that following a simulated handball game fatigue protocol, invertor and evertor muscles peak torque decreased. Additionally, a decrease in E:I muscles peak torque ratio was observed in the post-fatigue condition. The reduction in isokinetic maximum torque of the inverter and evertor muscles can be attributed to the effects of the fatigue protocol, as previously mentioned. These changes in muscle torques have implications for foot and ankle function. Lin and colleagues investigated the effects of inverter and evertor muscle fatigue and found that fatigue of these muscles leads to a decrease in ankle joint position sense in the frontal plane (35). Similarly, Rodriguez et al. demonstrated that fatigue disrupts the neuromuscular control of the evertor muscles (36). Bars Silva *et al.* reported that fatigue increases the time delay in muscle activation, which may be an underpinning mechanism contributing to the reduction in maximum torque of the inverter and evertor muscles (37). Sandrey and Kent observed that after fatigue, absolute error in joint position sense (an indicator of proprioceptive depth perception) increases. Castillo et al. found that fatigue of the inverter and evertor muscles results in decreased performance scores in functional tests, and impaired

balance control in athletes. Thus, fatigue-induced reductions in evertor muscle peak torque can decrease proprioceptive sensation and increase the risk of ankle sprains (38).

The results indicated that fatigue leads to a decrease in the E:I peak torque ratio. This decrease in the E:I peak torque ratio is associated with a higher risk of lateral ankle sprains (13). As shown, the decline in evertor muscle torque had greater contribution to decrease in E:I peak torque ratio than that of invertor muscles. It has been shown that prior to the occurrence of lateral ankle sprains, the E:I torque ratio decreases (13,39). In our study, fatigue was associated with decrease in evertor muscles peak torque at 60, 120, and 180 deg/s . However, the effect of fatigue on invertor muscles was different. Specifically, fatigue only decreased invertor muscle peak torque at 60 deg/s, with no effect at 120 and 180 deg/s. Based on these results, it seems that fatigue induced by a handball simulation protocol could contribute to an increased risk of lateral ankle sprains. The E:I peak torque ratio is derived from dividing the peak torque of the evertor muscles by that of the invertor muscles. Given that the reduction in evertor muscle torque is greater than that of the invertor muscles, the decrease in this ratio is likely due to the greater decline in evertor muscle torque. Therefore, special attention should be paid to the endurance capacity and strength of the evertor muscles during sports exercises to minimize the impact of fatigue and reduce the risk of lateral ankle sprains, which are among the most common injuries in handball players (6).

Conclusion

In general, the results of present study demonstrated that a handball match simulation-induced fatigue led to a decrease in the isokinetic peak torque of the

hamstring, quadriceps, invertor, and evertor muscles. Furthermore, the findings revealed that fatigue reduced both the H:Q peak torque ratio and the E:I peak torque ratio. These changes could contribute to increased risk of ACL tears and lateral ankle sprains. Considering that hamstring muscles were more affected in the reduction of the H:Q peak torque ratio, it is recommended to focus on strengthening and improving the endurance of this muscle in handball exercises. Additionally, due to the greater degrees of decrease in evertor muscles peak torque associated with the decrease in the E:I peak torque ratio, handball players are advised to prioritize enhancing the endurance and strength of the evertor muscles during their training sessions. Given that most lower limb injuries in handball occur in the final minutes of a match, it is recommended to specifically focus on training of hamstring and evertor muscles to increase their force production and endurance capacity to reduce injury risk and mitigate fatigue-related performance declines.

Acknowledgments

The authors thank all subjects who volunteered to participate in the present study.

Funding

This research did not receive any specific grant from funding agencies in the public, commercial, or not for profit sectors.

References

1. Póvoas SCA, Seabra AFT, Ascensão AAMR, Magalhães J, Soares JMC, Rebelo ANC. Physical and physiological demands of elite team handball. The Journal of Strength & Conditioning Research. 2012;26(12):3365–75.

2. Michalsik LB, Aagaard P. Physical demands in elite team handball: Comparisons between male and female players. J Sports Med Phys Fitness. 2015;55(9):878–91.

3. Engebretsen L, Soligard T, Steffen K, Alonso JM, Aubry M, Budgett R, et al. Sports injuries and illnesses during the London Summer Olympic Games 2012. British journal of sports medicine. 2013;47(7):407–14.

4. Michalsik LB. On-court physical demands and physiological aspects in elite team handball. Handball sports medicine: Basic science, injury management and return to sport. 2018;15–33.

5. Thorlund JB, Michalsik LB, Madsen K, Aagaard P. Acute fatigue-induced changes in muscle mechanical properties and neuromuscular activity in elite handball players following a handball match. Scandinavian journal of medicine & science in sports. 2008;18(4):462–72.

6. Langevoort G, Myklebust G, Dvorak J, Junge A. Handball injuries during major international tournaments. Scandinavian journal of medicine & science in sports. 2007;17(4):400–7.

7. Martín-Guzón I, Muñoz A, Lorenzo-Calvo J, Muriarte D, Marquina M, De la Rubia A. Injury prevalence of the lower limbs in handball players: A systematic review. International journal of environmental research and public health. 2021;19(1):332.

8. Edouard P, Chatard JC, Fourchet F, Collado H, Degache F, Leclair A, et al. Invertor and evertor strength in track and field athletes with functional ankle instability. Isokinetics and Exercise Science. 2011;19(2):91–6.

9. Ersoz M, Atalay NS, Kumbara F, Akyuz M. Investigation of effect of age, gender and limb dominance on ankle evertor/invertor peak torque ratios of healthy volunteers. Journal of Physical Therapy Science. 2009;21(3):263–7.

10. Aagaard P, Simonsen EB, Trolle M, Bangsbo J, Klausen K. Isokinetic hamstring/quadriceps strength ratio: influence from joint angular velocity, gravity correction and contraction mode. Acta Physiologica Scandinavica. 1995;154(4):421–7.

11. Sangnier S, Tourny-Chollet C. Comparison of the decrease in strength between hamstrings and quadriceps during isokinetic fatigue testing in semiprofessional soccer players. International journal of sports medicine. 2007;28(11):952–7.

12. van Melick N, van der Weegen W, van der Horst N. Quadriceps and hamstrings strength reference values for athletes with and without anterior cruciate ligament reconstruction who play popular pivoting sports, including soccer, basketball, and handball: a scoping review. journal of orthopaedic & sports physical therapy. 2022;52(3):142–55.

13. Pontaga I. Ankle joint evertor–invertor muscle torque ratio decrease due to recurrent lateral ligament sprains. Clinical Biomechanics. 2004;19(7):760–2.

14. Cozette M, Leprêtre PM, Doyle C, Weissland T. Isokinetic Strength Ratios: Conventional Methods, Current Limits and Perspectives. Front Physiol [Internet]. 2019;10. Available from: https://www.frontiersin.org/journals/physiology/articles/10.3389/fphys.2019.00567

15. Gleeson NP, Mercer TH. The utility of isokinetic dynamometry in the assessment of human muscle function. Sports medicine. 1996;21:18–34.

16. Aydoğ E, Aydoğ ST, Çakci A, Doral MN. Reliability of isokinetic ankle inversion-and eversion-strength measurement in neutral foot position, using the Biodex dynamometer. Knee Surgery, Sports Traumatology, Arthroscopy. 2004;12:478–81.

17. Fong DTP, Ha SCW, Mok KM, Chan CWL, Chan KM. Kinematics analysis of ankle inversion ligamentous sprain injuries in sports: five cases from televised tennis competitions. The American journal of sports medicine. 2012;40(11):2627–32.

18. Ameer MA. Kinematic comparison between single and double-leg jump landings in sagittal plane for male hand ball players. Turkish Journal of Sport and Exercise. 2016;18(3):108–15.

19. Bedo BLS, Pereira DR, Moraes R, Kalva-Filho CA, Will-de-Lemos T, Santiago PRP. The rapid recovery of vertical force propulsion production and postural sway after a specific fatigue protocol in female handball athletes. Gait & Posture. 2020;77:52–8.

20. Borg G. Borg's perceived exertion and pain scales. Human kinetics; 1998.

21. Cohen J. Statistical power analysis for the behavioral sciences. routledge; 2013.

22. Pinto MD, Blazevich AJ, Andersen LL, Mil-Homens P, Pinto RS. Hamstring-to-quadriceps fatigue ratio offers new and different muscle function information than the conventional non-fatigued ratio. Scandinavian journal of medicine & science in sports. 2018;28(1):282–93.

23. Massamba A, Hucteau E, Mallard J, Ducrocq GP, Favret F, Hureau TJ. Exercise-Induced Fatigue in Hamstring versus Quadriceps Muscles and Consequences on the Torque–Duration Relationship in Men. Medicine & Science in Sports & Exercise. 2022;54(12):2099–108.

24. Greco CC, Da Silva WL, Camarda SRA, Denadai BS. Fatigue and rapid hamstring/quadriceps force capacity in professional soccer players. Clinical physiology and functional imaging. 2013;33(1):18–23.

25. Greig M, Siegler JC. Soccer-specific fatigue and eccentric hamstrings muscle strength. Journal of athletic training. 2009;44(2):180–4.

26. Delextrat A, Baker J, Cohen DD, Clarke N. Effect of a simulated soccer match on the functional hamstrings-to-quadriceps ratio in amateur female players. Scandinavian journal of medicine & science in sports. 2013;23(4):478–86.

27. Greig M. The influence of soccer-specific fatigue on peak isokinetic torque production of the knee flexors and extensors. The American journal of sports medicine. 2008;36(7):1403–9.

28. Behrens M, Mau-Moeller A, Wassermann F, Bruhn S. Effect of fatigue on hamstring reflex responses and posterior-anterior tibial translation in men and women. PloS one. 2013;8(2):e56988.

29. Watanabe D, Wada M. Predominant cause of prolonged low-frequency force depression changes during recovery after in situ fatiguing stimulation of rat fast-twitch muscle. American Journal of Physiology-Regulatory, Integrative and Comparative Physiology. 2016;311(5):R919–29.

30. Allen DG, Lamb GD, Westerblad H. Impaired calcium release during fatigue. Journal of applied physiology. 2008;104(1):296–305.

31. Marshall PWM, Lovell R, Knox MF, Brennan SL, Siegler JC. Hamstring fatigue and muscle activation changes during six sets of Nordic hamstring exercise in amateur soccer players. The Journal of Strength & Conditioning Research. 2015;29(11):3124–33.

32. Bachasson D, Decorte N, Wuyam B, Millet GY, Verges S. Central and peripheral quadriceps fatigue in young and middle-aged untrained and endurance-trained men: A comparative study. Experimental Biology and Medicine. 2016;241(16):1844–52.

33. Chen TC, Lin KY, Chen HL, Lin MJ, Nosaka K. Comparison in eccentric exercise-induced muscle damage among four limb muscles. European journal of applied physiology. 2011;111:211–23.

34. Hamada T, Sale DG, MacDougall JD, Tarnopolsky MA. Interaction of fibre type, potentiation and fatigue in human knee extensor muscles. Acta physiologica scandinavica. 2003;178(2):165–73.

35. Delahunt E, Coughlan GF, Caulfield B, Nightingale EJ, Lin CWC, Hiller CE. Inclusion criteria when investigating insufficiencies in chronic ankle instability. Medicine & Science in Sports & Exercise. 2010;42(11):2106–21.

36. Rodrigues KA, Soares RJ, Tomazini JE. The influence of fatigue in evertor muscles during lateral ankle sprain. The Foot. 2019;40:98–104.

37. Silva BARS, Martinez FG, Pacheco AM, Pacheco I. Effects of the exercise-induced muscular fatigue on the time of muscular reaction of the fibularis in healthy individuals. Revista Brasileira de Medicina do Esporte. 2006;12:85–9.

38. Castillo GB, Brech GC, Luna NMS, Tarallo FB, Soares-Junior JM, Baracat EC, et al. Influence of invertor and evertor muscle fatigue on functional jump tests and postural control: A prospective cross-sectional study. Clinics. 2022;77:100011.

39. Kaminski TW, Hartsell HD. Factors contributing to chronic ankle instability: a strength perspective. Journal of athletic training. 2002;37(4):394.
