# Supplementary material for: Effect of simulated handball match-induced fatigue on isokinetic hamstring-to-quadriceps ratio and evertor-to-invertor ratio in professional players
Source: PLoS One. 2025 May 19;20(5):e0323978. doi: 10.1371/journal.pone.0323978 (PMC12088022; doi:10.1371/journal.pone.0323978)
Supplement: Attachment — Submitted filename: PONE-D-25-07828_R1_reviewer_reviewer comments.pdf [file pone.0323978.s003.pdf]

PLOS ONE

Effect of simulated handball match-induced fatigue on isokinetic hamstring-to-quadriceps ratio and evertor-to-invertor ratio in professional player

--Manuscript Draft--

**Manuscript Number:**	PONE-D-25-07828R1
**Article Type:**	Research Article
**Full Title:**	Effect of simulated handball match-induced fatigue on isokinetic hamstring-to-quadriceps ratio and evertor-to-invertor ratio in professional player
**Short Title:**	Effect of simulated handball match-induced fatigue on lower limb peak torques
**Corresponding Author:**	Hamed Esmaeili, Ph.D<br>University of Isfahan<br>Isfahan, Isfahan IRAN, ISLAMIC REPUBLIC OF
**Keywords:**	Isokinetic; Knee; ankle; fatigue; handball; hamstring-to-quadriceps ratio; torque; injury risk
**Abstract:**	Knee and ankle injuries are the most common injuries in the handball players. Many of these injuries occur towards the end of the game when fatigue sets in. The underlying mechanisms of this phenomenon are not well understood. This study investigated the effect of a handball match-simulation protocol on the hamstring-to-quadriceps (H:Q) and evertor-to-invertor (E:I) peak torque ratios. Thirty professional male handball players from Iranian super league teams participated in this study. Isokinetic concentric peak torque of the hamstrings, quadriceps, ankle invertor, and ankle evertors were measured at angular velocities of 60, 120, and 180 deg/s) before and after the simulated handball match-induced fatigue protocol using a Biodex Isokinetic Dynamometer. Peak torque ratio of H:Q and E:I were calculated and compared between pre- and post-fatigue conditions. Fatigue significantly reduced H:Q peak torque ratio at 60 (p=0.049), 120 (p=0.002) and 180 (p=0.014) deg/s, as well as E:I peak torque ratio at 60 (p=0.010), 120 (p=0.003) and 180 (p=0.003) deg/s. These changes could contribute to an increased risk of anterior cruciate ligament (ACL) tears and lateral ankle sprains. Given the greater reduction in the H:Q peak torque ratio in hamstring muscles, targeted strengthening and improving the endurance of these muscles is recommended for professional handball players to mitigate fatigue effects.
**Order of Authors:**	Ali Shirani Dastjerdi<br>Hamed Esmaeili, Ph.D<br>Morteza Sadeghi<br>Behzad Bashiri
**Response to Reviewers:**	Reviewer #1:<br>The purpose of this quasi-experimental study was to examine the effects of a functional fatigue protocol in handball on muscle group torque for the knee extensors and flexors and ankle invertors and evertors. There appeared to be a need for this study and the study purpose was clear. More information is needed in the methods section. There is some confusion in the presentation of the results, although figures and tables complement the narrative. More specific details are needed in the discussion and a limitations section is needed as well. A more developed presentation on central and peripheral fatigue is needed in the introduction which will serve a section of the discussion well. There were several word choice and word tense errors, as well as errors and inconsistencies in the reference section. See pdf for details comments and questions.<br>Thank you very much for your thorough evaluation and thoughtful suggestions. We greatly value your feedback, and we believe that the revisions we have made in response to your comments, especially in the methodology section, have significantly strengthened our manuscript. Below, we highlight the key changes implemented in response to your comments:<br>Regarding intermittent sport:<br>Intermittent sport, often referred to as intermittent exercise or interval training, involves alternating periods of high-intensity activity with periods of lower-intensity activity or

rest. So, Handball is an intermittent sport.
Your introduction to the concept of fatigue is superficial and appears to only apply to peripheral fatigue. Please more fully develop the concepts of peripheral and central fatigue first, then perhaps show the readers that the fatigue you are interested in mostly is related to peripheral fatigue. This needs to be well-referenced.
We have now expanded the introduction and discussion sections to include both peripheral and central fatigue.
We have also added the following to the manuscript:
Sample size calculation
Inclusion criteria
General warm up
A reference on the test-retest reliability of Borg scale measure
Peak torque per body mass considered for measurements
A brief description of the problem and the rationale/need for this study before restating the purpose
Expanded operational definitions of general and local fatigue in the discussion and introduction
Limitation section

Reviewer #2:
Thank you very much for your detailed review and constructive feedback. We appreciate your insights, which have helped strengthen our manuscript. The comments have been addressed and highlighted in the text. Please see our responses to each comment below.
1) The authors should justify why eccentric torque was not measured or acknowledge it as a limitation.
We have acknowledged this as a limitation in the manuscript.
2) Consider adding physiological fatigue markers (if available) to validate fatigue protocol
We have added physiological markers (Table 1) and expanded the discussion by adding the following:
“The proposed fatigue protocol was designed to induce high levels of physiological and psychological stress, ultimately leading to exhaustion. This was evident from the heart rate (HR) at exhaustion, which reached 186.31 ± 7.4 bpm, approximately 96% of the predicted maximum HR. Additionally, participants reported maximum values on the RPE score of 20, further confirming the extreme cardiovascular stress induced by the protocol. These findings, along with the observed reductions in quadriceps, hamstring invertor and evertor muscles peak torque per body mass and their respective ratios, strongly suggest that the protocol effectively induces fatigue. These results indicate that this protocol reliably simulates fatigue in handball players, making it a valuable tool for both training and research.”
3) Discuss whether the reduction in H:Q and E:I ratios reaches clinically significant thresholds
We have added the following to the discussion section:
“The large effect size (\eta_p^2 > 0.14) demonstrates that the simulated handball game fatigue protocol had a robust and clinically significant impact on knee and ankle musculature peak torques. This large effect size suggests that the simulated handball game fatigue protocol induced significant changes in muscle performance, as measured by peak torques. This is clinically relevant because peak torque is a key indicator of muscle strength and function, which are critical for athletic performance and injury prevention in handball players. For coaches and sports scientists, the large effect size underscores the importance of incorporating fatigue protocols into training regimens to better prepare athletes for the physical demands of competition. By replicating game-like fatigue conditions, athletes can develop greater resilience and adaptability in their knee and ankle musculature.”
4) if possible, provide effect size interpretations and confidence intervals
They have been added to the tables as suggested.
5) Ensure consistency in statistical reporting (rounding, decimal places, p-values)
We have reviewed the statistical reporting for consistency.
6) if possible, consider adding a sample size power analysis
It was added as suggested
7) Expand discussion with comparisons to other team sports
We have included compressions with soccer where it was relevant.
8) Provide practical training recommendations for injury prevention

Powered by Editorial Manager® and ProduXion Manager® from Aries Systems Corporation

	We have implemented these recommendations in the discussion.<br>9) You can benefit from the article https://doi.org/10.31459/turkjkin.1576269. in discussion section: When interpreting H:Q ratio reductions and their biomechanical consequences. or in introduction Section: To support the importance of the H:Q ratio in lower limb function.<br>Thank you so much for providing this valuable reference. We used this reference in the discussion section as suggested:<br>"Additionally, it has been shown that a decreased H:Q is associated with increased loading on the forefoot and toe regions (50). The increased loading could lead to increased risk of stress fracture in the forefoot region (51)."
**Additional Information:**	
**Question**	**Response**
**Financial Disclosure**<br><br>Enter a financial disclosure statement that describes the sources of funding for the work included in this submission. Review the submission guidelines for detailed requirements. View published research articles from *PLOS ONE* for specific examples.<br><br>This statement is required for submission and **will appear in the published article** if the submission is accepted. Please make sure it is accurate.<br><br>**Funded studies**<br>Enter a statement with the following details:<br>• Initials of the authors who received each award<br>• Grant numbers awarded to each author<br>• The full name of each funder<br>• URL of each funder website<br>• Did the sponsors or funders play any role in the study design, data collection and analysis, decision to publish, or preparation of the manuscript?<br><br>Did you receive funding for this work?	The author(s) received no specific funding for this work.
**Competing Interests**<br><br>Use the instructions below to enter a competing interest statement for this submission. On behalf of all authors, disclose any competing interests that could be perceived to bias this work—acknowledging all financial support and any other relevant financial or non-	The authors have declared that no competing interests exist.

Powered by Editorial Manager® and ProduXion Manager® from Aries Systems Corporation

financial competing interests.<br><br>This statement is required for submission and **will appear in the published article** if the submission is accepted. Please make sure it is accurate and that any funding sources listed in your Funding Information later in the submission form are also declared in your Financial Disclosure statement.<br><br>View published research articles from *PLOS ONE* for specific examples.<br><br>**NO authors have competing interests**<br><br>Enter: *The authors have declared that no competing interests exist.*<br><br>**Authors with competing interests**<br><br>Enter competing interest details beginning with this statement:<br><br>*I have read the journal's policy and the authors of this manuscript have the following competing interests: [insert competing interests here]*<br><br>* typeset	
**Ethics Statement**<br><br>Enter an ethics statement for this submission. This statement is required if the study involved:<br><br>• Human participants<br>• Human specimens or tissue<br>• Vertebrate animals or cephalopods<br>• Vertebrate embryos or tissues<br>• Field research<br><br>Write "N/A" if the submission does not require an ethics statement.<br><br>General guidance is provided below. Consult the submission guidelines for	Human Subject Research:<br>- institutional review board: Research Ethics Committee, University of Isfahan<br>- Approval Note from the Research Ethics Committee: the project was found to be in accordance with the ethical principles and the national norms and standards for conducting Medical Research in Iran.<br>Notice:<br>1. Although the proposal has been approved by the Biomedical Research Ethics Committee,<br>meeting the professional and legal requirements is the sole responsibility of the PI and other project collaborators.<br>2. This certificate is reliant on the proposal/documents received by this committee on 2022-08-<br>06. The committee must be notified by the PI as soon as the proposal/documents are modified.<br><br>-form of consent obtained: Written consent

<table>
<tr><td>

detailed instructions. **Make sure that all information entered here is included in the Methods section of the manuscript.**

Format for specific study types

Human Subject Research (involving human participants and/or tissue)

- Give the name of the institutional review board or ethics committee that approved the study
- Include the approval number and/or a statement indicating approval of this research
- Indicate the form of consent obtained (written/oral) or the reason that consent was not obtained (e.g. the data were analyzed anonymously)

Animal Research (involving vertebrate animals, embryos or tissues)

- Provide the name of the Institutional Animal Care and Use Committee (IACUC) or other relevant ethics board that reviewed the study protocol, and indicate whether they approved this research or granted a formal waiver of ethical approval
- Include an approval number if one was obtained
- If the study involved *non-human primates*, add *additional details* about animal welfare and steps taken to ameliorate suffering
- If anesthesia, euthanasia, or any kind of animal sacrifice is part of the study, include briefly which substances and/or methods were applied

Field Research

Include the following details if this study involves the collection of plant, animal, or other materials from a natural setting:

- Field permit number
- Name of the institution or relevant body that granted permission

</td><td></td></tr>
<tr><td>

Data Availability

Authors are required to make all data underlying the findings described fully available, without restriction, and from the time of publication. PLOS allows rare

</td><td>Yes - all data are fully available without restriction</td></tr>
</table>

exceptions to address legal and ethical concerns. See the PLOS Data Policy and FAQ for detailed information.<br><br>A Data Availability Statement describing where the data can be found is required at submission. Your answers to this question constitute the Data Availability Statement and **will be published in the article**, if accepted.<br><br>**Important:** Stating 'data available on request from the author' is not sufficient. If your data are only available upon request, select 'No' for the first question and explain your exceptional situation in the text box.<br><br>Do the authors confirm that all data underlying the findings described in their manuscript are fully available without restriction?	
**Describe where the data may be found in full sentences. If you are copying our sample text, replace any instances of XXX with the appropriate details.**<br><br>• If the data are **held or will be held in a public repository**, include URLs, accession numbers or DOIs. If this information will only be available after acceptance, indicate this by ticking the box below. For example: *All XXX files are available from the XXX database (accession number(s) XXX, XXX.*).<br>• If the data are all contained **within the manuscript and/or Supporting Information files**, enter the following: *All relevant data are within the manuscript and its Supporting Information files.*<br>• If neither of these applies but you are able to provide **details of access elsewhere**, with or without limitations, please do so. For example:<br><br>*Data cannot be shared publicly because of [XXX]. Data are available from the XXX Institutional Data Access / Ethics Committee (contact via XXX) for researchers who meet the criteria for*	NA

access to confidential data.<br><br>*The data underlying the results presented in the study are available from (include the name of the third party and contact information or URL).*<br>• This text is appropriate if the data are owned by a third party and authors do not have permission to share the data.<br><br>* typeset	
Additional data availability information:	Tick here if your circumstances are not covered by the questions above and you need the journal's help to make your data available.; Tick here if the URLs/accession numbers/DOIs will be available only after acceptance of the manuscript for publication so that we can ensure their inclusion before publication.

Powered by Editorial Manager® and ProduXion Manager® from Aries Systems Corporation

Manuscript

Click here to access/download;Manuscript;Fin Clear Manuscript.docx

Effect of simulated handball match-induced fatigue on isokinetic hamstring-to-quadriceps ratio and evertor-to-invertor ratio in professional players

Ali Shirani Dastjerdi[1], Hamed Esmaeili[1*], Morteza Sadeghi[1], Behzad Bashiri[2]

[1] Department of Sport Injuries and Corrective Exercises, Faculty of Sport Sciences, University of Isfahan, Isfahan, Iran

[2] Department of Biomedical Engineering, Faculty of Engineering, International University of Science and Technology in Kuwait, Ardiya, Kuwait

Short Title: Effect of simulated handball match-induced fatigue on lower limb peak torques

* Corresponding author: Hamed Esmaeili

Email: H.esmaeili@spr.ui.ac.ir

Abstract

Knee and ankle injuries are the most common injuries in handball players. Many of these injuries occur toward the end of the game when fatigue sets in. The underlying mechanisms of this phenomenon are not well understood. This study investigated the effect of a handball match-simulation protocol on the hamstring-to-quadriceps (H:Q) and evertor-to-invertor (E:I) peak torque ratios. Thirty professional male handball players from Iranian super league teams participated in this study. Isokinetic concentric peak torque of the hamstrings, quadriceps, ankle invertor, and ankle evertors were measured at angular velocities of 60, 120, and 180 deg/s) before and after the simulated handball match-induced fatigue protocol using a Biodex Isokinetic Dynamometer. Peak torque ratio of H:Q and E:I were calculated and compared between pre- and post-fatigue conditions. Fatigue significantly reduced H:Q peak torque ratio at 60 ($p=0.049$), 120 ($p=0.002$) and 180 ($p=0.014$) deg/s, as well as E:I peak torque ratio at 60 ($p=0.010$), 120 ($p=0.003$) and 180 ($p=0.003$) deg/s. These changes could contribute to an increased risk of anterior cruciate ligament (ACL) tears and lateral ankle sprains. Given the greater reduction in the H:Q peak torque ratio in hamstring muscles, targeted strengthening and improving the endurance of these muscles is recommended for professional handball players to mitigate fatigue effects.

Keywords: Isokinetic, Knee, Ankle, Fatigue, Handball, Hamstring-to-quadriceps ratio, Torque, Injury risk

Introduction

Handball, as a complex and highly demanding intermittent sport, involves multiple high-intensity runs, frequent body contact, and other high-intensity actions to overcome opponents (1). A handball match involves a large number of repeated accelerations, sprints, jumps, blocking, pushing and rapid changes in moving directions (2). Sudden changes of direction, decelerations and accelerations, vertical and horizontal jumps, throws, thrusts, pulls, and powerful, fast-paced clashes are among the movements of handball sport that have made it a sport with high risk of injury (3).

These physical demands are expected to lead to fatigue, which could be at least partially responsible for the reduction in the quantity and quality of high-intensity actions toward the end of a match compared to the beginning (4). Fatigue can be categorized into two types: central fatigue, which refers to a decline in performance due to factors occurring near the neuromuscular synapses, and peripheral fatigue, characterized by a reduction in muscle contraction strength caused by processes further from these synapses (5–7). Both forms of fatigue can appear during training sessions or matches, potentially leading to impaired coordination, muscle mechanical properties and neuromuscular activity, and a higher frequency of mistakes (8). During elite male handball matches, there was a decrease in high-intensity running time, demanding actions, and time spent with a heart rate above 80% of maximum from the first half to the second (9). Metabolic markers such as plasma free fatty acids, glycerol, glucose, and uric acid increased during the first half, with further increases in free fatty acids and glycerol during the second half (9). These changes led to decline in sprint performance and reduction in vertival jump height compared to baseline levels, indicating significant fatigue development (9). Moreover, handball match-induced fatigue has been shown to increase center of

pressure area during landing and decrease maximum propulsion force for countermovement jump (10).

It has been shown that players who play 70 percent of the total match time experience changes such as reduction in high-intensity running, reduction in heart rate, reduction in technical and impaired physical performance, resulting from fatigue (11). This condition could increase demand on musculoskeletal system and leaves players at high risk of injury. Notably, the highest number of injuries during six periods of international competitions occurred between minutes 16 to 30 of the first half and 46 to 60 of the second half (12).

Given these findings, it can be suggested that physical fatigue deteriorates neuromuscular and sensorimotor control, which may elevate the risk of injury. Handball players report a higher prevalence of lower limb injuries than injuries to the upper limbs, neck, or head (12,13). For instance, approximately 30% of injuries occur in the knee, and 25% in the ankle (13).

The hamstring-to-quadriceps (H:Q) peak torque ratio in the knee joint and evertor to invertor (E:I) peak torque ratio in the ankle are two risk factors used to assess muscle balance or imbalance in the knee and ankle (14–17). Deterioration in these ratios can increase the risk of injury. A decreased H:Q ratio implies that the quadriceps are disproportionately stronger than the hamstrings, which may stress the anterior cruciate ligament (ACL) and may lead to injuries (18). A decrease in the E:I peak torque ratio is linked to a higher risk of lateral ankle sprains (19).

Isokinetic muscle peak torque is a critical metric in assessing injury risk, particularly in sports and rehabilitation settings. Isokinetic testing can identify neuromuscular deficits, such as reduced torque production or delayed muscle activation, which are often linked to injury risk (20,21). For example, a decline in hamstring peak torque

has been associated with an increased risk of hamstring strains in soccer players (21).

Given the high incidence of lower limb injuries among handball players, particularly when players are fatigued during matches, it is crucial to investigate the effects of match-induced fatigue on H:Q and E:I peak torque ratios in handball. The aim of this study was to evaluate the effects of simulated handball match-induced fatigue on the isokinetic hamstring-to-quadriceps and invertor-to-evertor ratios in professional handball players.

Materials and Methods

Participants

Thirty professional male handball players from Iranian Super League teams (age: 27 ± 3.1 years; height: 186.9 ± 7.3 cm; mass: 86.8 ± 6.7 kg) participated in this study. A prior power analysis revealed that for a statistical power of 0.9 at an alpha level of 0.05 and an effect size of 0.41, a sample size of at least 26 participants is required (22). The effect size was chosen from a previous study that had used a similar fatigue protocol (23). Participant recruitment for this study began on August 15, 2023, and concluded on November 30, 2023. The inclusion criteria for participation required all subjects to have engaged in competitive handball for a minimum of 5 years and to participate in more than thee training sessions per week. During the preseason, the players completed 8–10 training sessions per week, while during the season, they attended 5–7 sessions per week. All participants were active team members and participated in every match. Volunteers who reported injuries in the lower limbs in the last 6 months were excluded. All participants provided written informed consent. This study was approved by the University of Isfahan Ethics Committee and conducted in accordance with the Declaration of Helsinki.

Testing preparation

Initially, participants were briefed on the testing procedure. The participants began with an 8-minute warmup on a stationary bicycle with no resistance at moderate velocity (70-80 revolutions per minute) (24). Next, they sat on the dynamometer chair (Biodex Multi-joint System 3, Biodex Medical Systems, USA) which was adjusted so they had free and full movement in knee and ankle joints. Participants were fixed with two horizontal and vertical belts, and the test limb was fixed to the arm of the device. The seat of the device was adjusted to an angle of 100 degrees. The arm of the device was adjusted to a tilit angle of 0 deg. The movement axis of the knee was aligned parallel to the movement axis of the device. For the ankle test, the participant's ankle was positioned on the footplate and the foot was secured using two Velcro straps.

For each test session, the range of motion of ankle and knee joints was determined first (25). Concentric isokinetic peak torque for quadriceps, hamstring, ankle invertors, and ankle evertors was measured at three different angular velocities of 60, 120 and 180 deg/s by a Biodex Multi-joint System 3 dynamometer (Biodex Medical Systems, USA) on the landing leg in a random order. The landing leg was defined as the leg that players use to land after jumping (26,27). Each angular velocity was measured with one set of six repetitions. In each test, participants were instructed to perform the movements with maximum effort. Verbal encouragement was provided during the test. Peak torques were assessed at baseline and immediately after the handball-specific fatigue protocol to examine its effects on the outcomes.

Fatiguing protocol

A previously used fatigue protocol was utilized to simulate match-induced fatigue (10) which included special movements during a handball match, such as sprinting, jumping, landing, sidestep cutting, and lateral and backward shifting (Fig 1, caption for more details). The protocol was divided into gradually increasing rounds, requiring participants to complete each round as quickly as possible. The number of rounds corresponded to the number of laps in the circuit (e.g., round I: 1 lap; round II: 2 laps; and so on). For each round, time of effort, heart rate using Polar F4 Blue ICE, and the rate of perceived exertion (RPE) using the 15-point Borg scale with a a range of 6-20 were recorded (28). In this Borg Scale, a score of six indicates no fatigue, while scores between 17 and 20 indicate the inability to continue the exercises. During the trials, if participants reported a score of 17 or higher at the end of a round, the fatigue protocol ended. If they reported a lower score, they performed another round until they reached a score of 17. Borg scale has been shown to have high test-retest reliability (29,30).

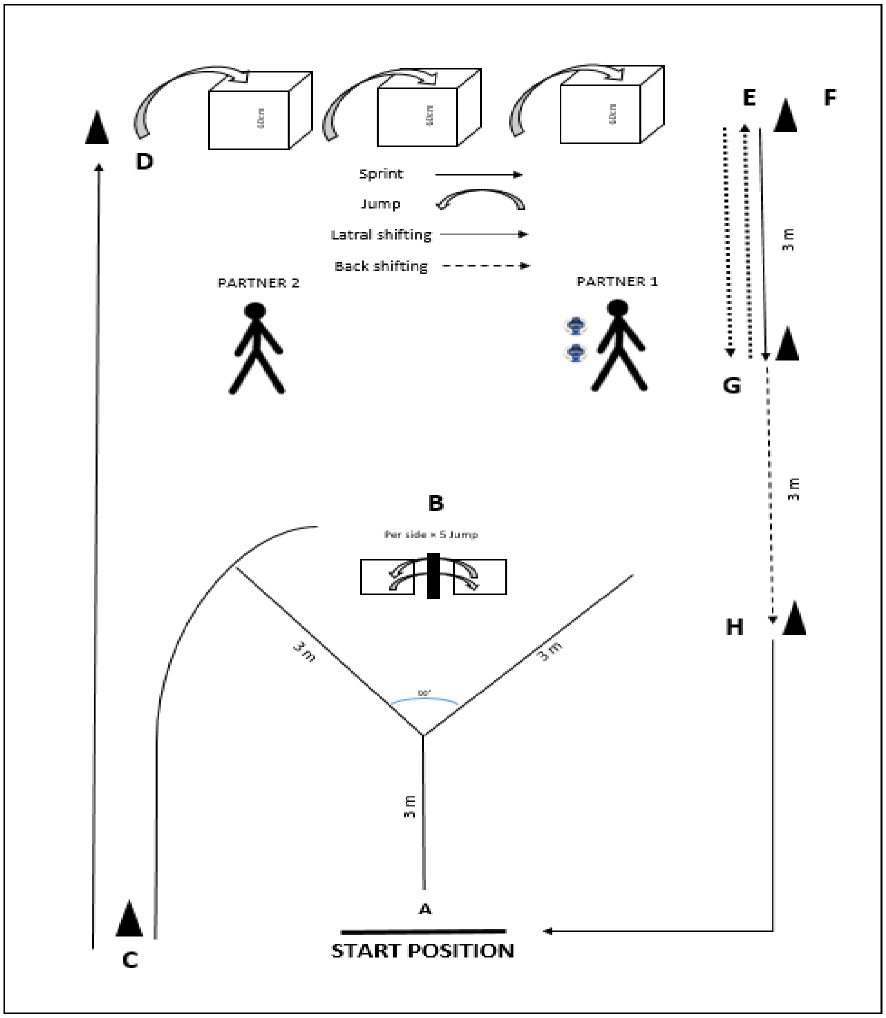

Figure 1. Illustration of the fatigue protocol based on specific movements performed by handball athletes. The athletes performed the following actions in all of the stations as fast as possible: (A) Acceleration and sidestep cutting maneuver for the right and run until the end of the 3-m distance; receive a pass (at the end of the 3-m length) from a partner positioned 2.5 m away (Partner 1); backshift until the 90°; run 3 m for the left side, and perform an accurate throw to a partner positioned 2.5 m away (Partner 2); this task was performed twice per lap; (B) Ten double-leg lateral jumping/landing over a barrier (20-cm high), performed as fast as possible; (C) Sprint; (D) Double-leg plyometric jumps, performed as fast as possible; (E) Lateral shifting; (F) Sprint; (G) Back Shifting; and (H) Sprint with a 90° change of direction. The protocol was divided into rounds, where the round number represents the number of laps in the circuit (e.g., round I: 1 lap, round II: 2 laps, round III: 3 laps, and so on) until the participant is unable to continue performing the protocol.

Data analysis

Peak knee flexion, knee extension, ankle inversion and ankle eversion torque were recorded for each repetition. Between each set of the test, participants rested for 2-

min. To calculate the H:Q ratio, peak flexion torque was divided by the peak extensor torque [15 Bilateral]. To calculate the E:I ratio, peak eversion torque was divided by the peak inversion torque (19). Peak torques per body mass, along with the peak torque ratios of H:Q and E:I, were used in subsequent analyses.

Statistical Analysis

Shapiro-Wilk test was used to ensure normality of data distribution. To investigate the effect of fatigue on the outcomes, a 2 (condition: pre- and post-fatigue) × 3 (angular velocity: 60, 120, and 180 deg/s) repeated measures ANOVA was used. Holm-Bonferroni adjustment was used for multiple comparisons. Effect size (ES), reported using partial eta squared, η_p^2, was classified as small (η_p^2 = 0.01), medium (η_p^2 = 0.06), and large (η_p^2 = 0.14) (31). All statistical analyses were performed at a significance level of $p < 0.05$ using SPSS software, version 18.0 (SPSS Inc, Chicago IL, USA).

Results

Fatigue Markers

Each participant executed a particular number of laps to achieve fatigue, ranging between four and eleven (5.8 ± 2.4 laps or 276 ± 79.2 s). Significant increases were observed for heart rate ($F_{(1,29)} = 341.58$; $p < 0.001$) and RPE ($F_{(1,29)} = 392.42$; $p < 0.001$) (table 1).

Table 1. Mean and standard deviation (±) of the fatigue parameters.

	Baseline	Fatigue State	F	P
**Heart rate**	64.26 ± 8.2	186.31 ± 7.4	341.58	**< 0.001**
**RPE**	6 ± 0	20 ± 0	392.42	**< 0.001**

Bold values indicate significant difference in p ≤ 0.05.

Effect of fatigue on peak torques

The results showed that the main effect of fatigue ($F_{(1,29)} = 36.14$, $p < 0.01$, ES = 0.56) and the main effect of angular velocity ($F_{(2,58)} = 87.58$, $p < 0.01$, ES = 0.751) were significant, but the interaction effect was not significant ($F_{(2,58)} = 2.20$, p = 0.120, ES = 0.070) on the hamstring peak torque to body mass ratio. Multiple comparisons showed that fatigue decreased hamstring peak torque in all angular velocities (Table 2).

For the quadriceps peak torque to body mass ratio, the results showed significant main effects for fatigue ($F_{(1,29)} = 7.18$, p = 0.012, ES = 0.198) and angular velocity ($F_{(2,58)} = 296.68$, p = 0.001, ES = 0.911), and a significant fatigue × angular velocity interaction effect ($F_{(2,58)} = 4.20$, p = 0.020, ES = 0.127). This indicates that fatigue affects quadriceps peak torque differently at various angular velocities. Post-hoc tests revealed that fatigue was associated with a decrease in quadriceps peak torque at 60 deg/s and 180 deg/s, but not at 120 deg/s (Table 2).

Table 2. Comparison of hamstring and quadriceps peak torque ratio (in Nm/body mass) in the pre- and post-fatigue at different angular velocities. Data are presented in Mean ± SD.

		Pre	Post	Mean Difference	Standard Error	95% Confidence Interval	t	p_{holm}
Hamstring	60 deg/s	1.83 ± .36	1.56 ± .25	0.27333	0.0649	(0.476 - 1.330)	4.214	**0.001**
	120 deg/s	1.55 ± .24	1.43 ± .26	0.12247	0.0360	(0.280 - 1.077)	3.398	**0.006**
	180 deg/s	1.32 ± .23	1.18 ± .23	0.13907	0.0198	(0.823 - 1.808)	7.031	**0.001**
Quadriceps	60 deg/s	2.84 ± .35	2.65 ± .43	0.1827	0.0649	(0.186 - 0.961)	2.81	**0.026**
	120 deg/s	2.33 ± .34	2.24 ± .31	0.0852	0.0564	(-0.092 - 0.638)	1.51	0.141
	180 deg/s	1.68 ± .25	1.60 ± .27	0.9749	0.0668	(0.036 - 0.772)	2.19	**0.041**

Bold values indicate significant difference in $p \leq 0.05$.

Regarding the invertor peak torque to body mass ratio, the results of ANOVA showed a significant main effect of fatigue ($F_{(1,29)} = 12.97$, p = 0.001, ES = 0.309) and angular velocity ($F_{(2,58)} = 71.46$, p = 0.001, ES = 0.711), but no significant fatigue × angular velocity interaction ($F_{(2,58)} = 2.11$, p = 0.130, ES = 0.068). Multiple comparisons showed that, after fatigue, invertor peak torque decreased only in 60 deg/s (Table 3).

Repeated measures analysis of variance showed significant main effects of fatigue ($F_{(1,29)} = 44.01$, $p = 0.001$, ES = 0.603) and angular velocity ($F_{(2,58)} = 99.48$, $p = 0.001$, ES = 0.774), and a significant fatigue × angular velocity interaction ($F_{(2,58)} = 3.35$, $p = 0.042$, ES = 0.104) on the evertor peak torque to body mass ratio. The handball-specific fatigue protocol was associated with decrease in evertor peak torque in all three angular velocities (Table 3).

Table 3. Comparison of invertor and evertor peak torque ratio (in Nm/body mass) in the pre- and post-fatigue at different angular velocities. Data are presented in Mean ± SD.

		Pre	Post	Mean Difference	Standard Error	95% Confidence Interval	t	p_{holm}
**Invertor**	60 deg/s	0.44 ± .10	0.39 ± .10	0.0469	.01233	(0.290 - 1089)	3.80	**.003**
	120 deg/s	0.37 ± .08	0.35 ± .09	0.0240	.01194	(-0.006 - 0.734)	2.01	.161
	180 deg/s	0.31 ± .09	0.30 ± .06	0.0165	.01179	(-0.111 – 0.617)	1.40	.345
**Evertor**	60 deg/s	0.42 ± .12	0.33 ± .08	0.08779	.01389	(0.684 – 1.612)	6.319	**.001**
	120 deg/s	0.35 ± .09	0.27 ± .06	0.07345	.01292	(0.586 – 1.478)	5.685	**.001**
	180 deg/s	0.27 ± .08	0.21 ± .05	0.05667	.01202	(0.435 – 1.276)	4.714	**.001**

Bold values indicate significant difference in $p \leq 0.05$.

Effect of fatigue on peak torque ratios

ANOVA test showed significant main effects of fatigue ($F_{(1,29)} = 18.143$, $p = 0.001$, ES = 0.385) and angular velocity ($F_{(2,58)} = 60.019$, $p = 0.001$, ES = 0.674), but no significant fatigue × angular velocity interaction effect ($F_{(2,58)} = 0.526$, $p = 0.594$, ES = 0.018) on H:Q peak torque ratio. Fig 2 illustrates H:Q peak torque ratio in the pre- and post-fatigue in different angular velocities. As shown, fatigue decreased H:Q ratio in 60 ($t_{(1,29)} = 2.662$, $p = 0.049$), 120 ($t_{(1,29)} = 4.202$, $p = 0.002$) and 180 ($t_{(1,29)} = 3.327$, $p = 0.014$) deg/s.

ANOVA test showed significant main effects for fatigue ($F_{(1,29)} = 17.39$, $p = 0.001$, ES = 0.375) and angular velocity ($F_{(2,58)} = 29.90$, $p = 0.001$, ES = 0.508), but no significant fatigue × angular velocity interaction effect ($F_{(2,58)} = 1.63$, $p = 0.206$, ES = 0.053) on E:I. As shown in Fig 3, fatigue decreased E:I in 60 ($t_{(1,29)} = 3.469$, $p = 0.010$), 120 ($t_{(1,29)} = 4.164$, $p = 0.003$) and 180 ($t_{(1,29)} = 4.198$, $p = 0.003$) deg/s.

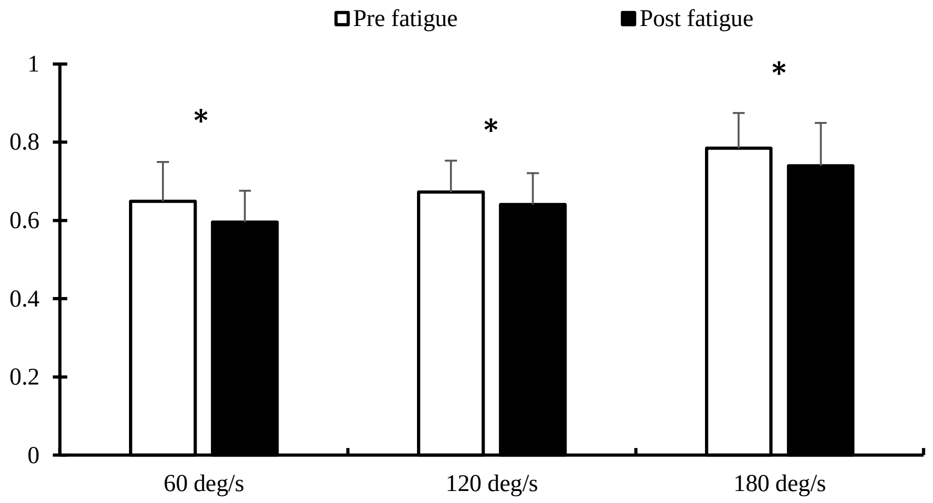

Fig 2. Comparison of H:Q peak torque ratio at different angular velocities in pre- and post-fatigue. (*) stands for significant difference in $p \leq 0.05$.

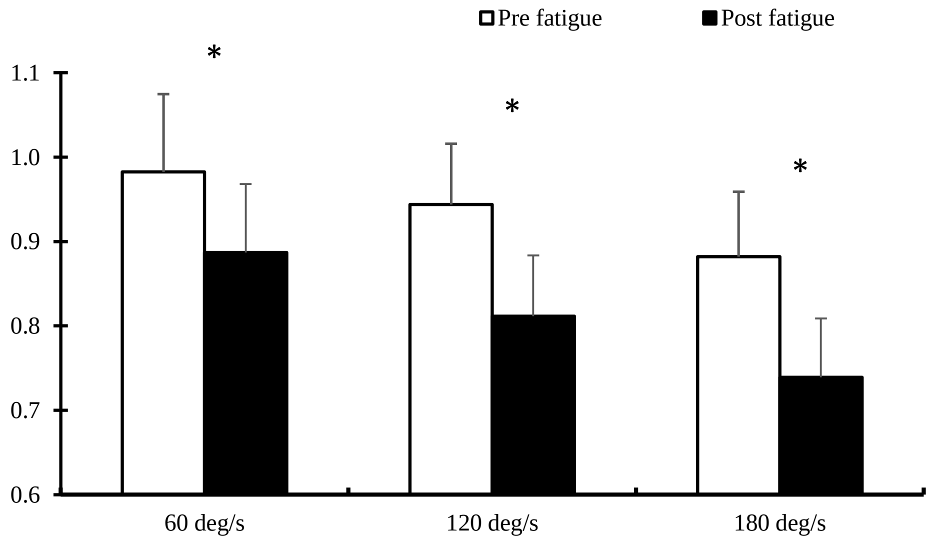

Fig 3. Comparison of E:I peak torque ratio in different angular velocities in pre-and-post fatigue. (*) stands for significant difference in $p \leq 0.05$.

Discussion

Knee and ankle injuries are the most common injury sites among handball players, especially when players experience fatigue during matches. The effect of match-

induced fatigue on hamstring, quadriceps, ankle invertors and evertors, and their subsequent ratios is not well understood. The aim of the current study was to investigate the effect of simulated handball match-induced fatigue on H:Q and E:I peak torque ratios in Iranian handball Super League players. Our results showed that fatigue decreased peak hamstring and quadriceps muscles torque per body mass and the H:Q peak torque ratio. Also, the results showed that fatigue was associated with a decrease in peak invertor and evertor muscles torque per body mass and decrease in E:I peak torque ratio. In line with our results, Pinto *et al*. and Sangnier and Tourny-Chollet showed decreases in knee flexor and extensor torque and hamstring-to-quadriceps ratios (17,32). Pinto et al showed that after completing a 30-repetition isokinetic fatigue test protocol in professional soccer players, the knee extensor and flexor moments decreased and the H:Q was decreased at the end of the test due to a significant reduction in knee flexor moment as well (32). Sangnier and Tourny-Chollet also using an isokinetic fatigue protocol on professional soccer players showed decreased strength of hamstring and quadriceps muscles (17). Massamba *et al*. showed that after a 5-min all-out exercise training-induced fatigue, the maximum voluntary isometric force of quadriceps and hamstring muscles of healthy men decreased (33). Our observed decreases in hamstring and quadriceps muscle peak torques in the current study, indicate effect of a simulated handball match-induced fatigue on knee flexor and extensor muscles strength and force output. While it has been well known that fatigue affects peak force output, training modalities seem to have different effects on force output reduction (34–38). After completion of different fatigue protocols, similar reductions in force output are observed in knee flexor and extensor muscles concentric or isometric measurements (33,35). The decrease in strength and torque output may result from a reduced ability of the muscles to generate force, which is associated with a decrease in the number of cross-bridges generating force and the number of cross-bridges actively working in

the muscle (39). This reduction can result from either general or localized muscle fatigue. Fatigue has both central and peripheral origins (40). Peripheral fatigue is characterized by inability of the muscle to contract effectively (41). Central fatigue, on the other hand, involves a significant reduction in the neural drive to the muscles (40), typically occuring after prolonged efforts. Therefore, it is plausible that the fatigue induced by our protocol has peripheral origins. However, according to the central governor model (42), the central component may also play a role in this type of exercise. This model suggests that the central nervous system interacts with the muscles to protect vital organs (e.g., the heart and brain) by reducing neural drive in high-demand situations, even during short-term exercises (43). Thus, the fatigue protocol used in the present study may induce both peripheral and central fatigue.

The proposed fatigue protocol was designed to induce high levels of physiological and psychological stress, ultimately leading to exhaustion. This was demonstrated by the heart rate (HR) recorded at the point of exhaustion, which reached 186.31 ± 7.4 bpm, approximately 96% of the predicted maximum HR. Additionally, participants reported maximum values on the RPE score of 20, further confirming the extreme cardiologic stress induced by the protocol. These findings, along with the observed reductions in quadriceps, hamstring invertor and evertor muscles peak torque per body mass and their respective ratios, strongly suggest that the protocol effectively induces fatigue. These results indicate that this protocol reliably simulates fatigue in handball players, making it a valuable tool for both training and research purposes.

Our results showed that knee flexor muscles are influenced by handball match induced fatigue as well as knee extensors. Changes in fatigued condition are repetitively reported for knee extensor muscles (44,45). However, our results are inconsistent with those of Marshal *et al.* who showed soccer simulation match-

induced fatigue had no effect on hamstring muscles peak torque (44). This inconsistency could be attributed to differences in the population and fatigue protocol. Marshal *et al.* studied soccer players and a soccer simulation match, whereas the current study included professional handball players and used a handball match-simulating protocol which engaged both hamstring and quadriceps muscles.

Our results showed that H:Q peak torque ratio decreased after fatigue protocol. The reduction in this ratio was associated with a decrease in hamstring and quadriceps muscles peak torques. Since this ratio is calculated by dividing hamstring peak torque by quadriceps peak torque, it seems that hamstring muscles are more affected by fatigue. Pinto *et al.* showed that after a fatigue protocol, hamstring muscles torque decreased to a greater extent than that of quadriceps (46), which is in line with the results of our study. Hamstring muscles besides their complex muscular architecture and multi-joint function, comprise a significant number of type II fibers and are less utilized in most daily activities, making them more susceptible to muscle damage and fatigue (47,48). Although the mechanism of hamstring and ACL injuries is still a subject of debate, it is well-established that neuromuscular fatigue is an important risk factor (49). For example, changes in hamstring muscle coordination, alterations in hamstring length during late swing phase of running cycle, and altered ACL-hamstring reflex are associated with muscle stiffness regulation and knee stability after a fatigue protocol (32). Additionally, it has been shown that a decreased H:Q is associated with increased loading on the forefoot and toe regions (50). The increased loading could lead to increased risk of stress fracture in the forefoot region (51). Overall, these findings also indicate that knee flexors should be well-trained to enhance their force production capacity and endurance, thus reducing susceptibility to hamstring and ACL injuries. Additionally, a reduction in the H:Q peak torque ratio increases the risk of ACL injury (52).

The results of the present study demonstrated that following a simulated handball game fatigue protocol, invertor and evertor muscles peak torque decreased. Additionally, a decrease in E:I muscles peak torque ratio was observed in the post-fatigue condition. The reduction in isokinetic maximum torque of the invertor and evertor muscles can be attributed to the effects of the fatigue protocol, as previously mentioned. These changes in muscle torques have implications for foot and ankle function. Lin and colleagues, using an isokinetic concentric-eccentric fatigue protocol, investigated the effects of invertor and evertor muscles fatigue and found that it leads to a decrease in ankle joint position sense in the frontal plane (53). Similarly, Rodriguez et al. demonstrated that after inducing fatigue by maintained eversion and plantar flexion at 70% of the maximum voluntary isometric contraction until a 10% decline in force, neuromuscular control was compromised in fatigued evertor muscles (54). Bars Silva *et al.* induced fatigue through active localized ankle eversion exercises against resistance and demonstrated that exercise-induced muscle fatigue significantly increases the reaction time of the peroneal muscles (55). This delayed reaction time can impair the muscles' ability to respond quickly to sudden changes in ankle position, potentially increasing the risk of ankle injuries (55). Sandrey and Kent used a fatigue protocol targeting the ankle evertors, followed by tests to measure their ability to detect and replicate specific ankle positions in the frontal plane (56). They also assessed absolute error in joint position sense, as an indicator of proprioceptive depth perception; they demonstrated that eversion fatigue negatively affects the ankle's ability to sense its position in the frontal plane (56). Castillo et al. found that fatigue in the invertor and evertor muscles significantly impaired performance in functional jump tests (57). They reported that participants experienced reduced jump height, distance, and control after muscle fatigue, indicating a decline in dynamic stability and power output (57). Thus, fatigue-

induced reductions in evertor muscle peak torque can decrease proprioceptive sensation and increase the risk of ankle sprains

Our results indicate that fatigue led to a decrease in the E:I peak torque ratio. This decrease in the E:I peak torque ratio is associated with a higher risk of lateral ankle sprains (19). As shown, the decline in evertor muscle torque had greater contribution to decrease in E:I peak torque ratio than that of invertor muscles. It has been shown that prior to the occurrence of lateral ankle sprains, the E:I torque ratio decreases (19,58). In our study, fatigue was associated with decreases in evertor muscles peak torque at 60, 120, and 180 deg/s . However, the effect of fatigue on invertor muscles was different. Specifically, fatigue only decreased invertor muscle peak torque at 60 deg/s, with no effect at 120 and 180 deg/s. Based on these results, it seems that fatigue induced by a handball simulation protocol could contribute to an increased risk of lateral ankle sprains. The E:I peak torque ratio is derived from dividing the peak torque of the evertor muscles by that of the invertor muscles. Given that the reduction in evertor muscle torque is greater than that of the invertor muscles, the decrease in this ratio is likely due to the greater decline in evertor muscle torque. Therefore, special attention should be given to the endurance capacity and strength of the evertor muscles during sports exercises to minimize the impact of fatigue and reduce the risk of lateral ankle sprains, which are among the most common injuries in handball players (12).

The large effect size ($\eta_p^2 > 0.14$) demonstrates that the simulated handball game fatigue protocol had a robust and clinically significant impact on knee and ankle musculature peak torques. This large effect size suggests that the simulated handball game fatigue protocol induced significant changes in muscle performance, as measured by peak torques. This is clinically relevant because peak torque is a key indicator of muscle strength and function, which are critical for athletic performance

and injury prevention in handball players. For coaches and sports scientists, the large effect size underscores the importance of incorporating fatigue protocols into training regimens to better prepare athletes for the physical demands of competition. By replicating game-like fatigue conditions, athletes can develop greater resilience and adaptability in their knee and ankle musculature.

Despite the promising findings of this study, some limitations should be acknowledged. First, we limited the measurements to concentric contrations to calculate conventional ratios. However, a previous study have shown that the behavior of eccentric torque is similar to that of concentric in terms of conventional ratios (59). Second, we were unable to differentiate between central and peripheral causes of fatigue. Future studies should consider incorporating methods to identify the causes of fatigue within this type of protocol. Lastly, the conditions of handball match differ from those created in the simulation protocol used in this study. In handball, many injuries and injury-related situations result from collisions between players, which were not accounted for in the current study. However, this study focused on examining intrinsic factors within individuals identifying intrinsic mechanisms.

Conclusion

In general, the results of present study demonstrated that a handball match simulation-induced fatigue led to a decrease in the isokinetic peak torque of the hamstring, quadriceps, invertor, and evertor muscles. Furthermore, the findings revealed that fatigue reduced both the H:Q peak torque ratio and the E:I peak torque ratio. These changes could contribute to increased risk of ACL tears and lateral ankle sprains. Considering that hamstring muscles were more affected in the reduction of the H:Q peak torque ratio, it is recommended to focus on strengthening and improving the endurance of this muscle in handball exercises. Additionally, due to

the greater degrees of decrease in evertor muscles peak torque associated with the decrease in the E:I peak torque ratio, handball players are advised to prioritize enhancing the endurance and strength of the evertor muscles during their training sessions. Given that most lower limb injuries in handball occur in the final minutes of a match, it is recommended to specifically focus on training of hamstring and evertor muscles to increase their force production and endurance capacity to reduce injury risk and mitigate fatigue-related performance declines.

Acknowledgments

The authors thank all subjects who volunteered to participate in the present study.

Funding

This research did not receive any specific grant from funding agencies in the public, commercial, or not for profit sectors.

References

1. Póvoas SCA, Seabra AFT, Ascensão AAMR, Magalhães J, Soares JMC, Rebelo ANC. Physical and physiological demands of elite team handball. J Strength Cond Res. 2012;26(12):3365–75.
2. Michalsik LB, Aagaard P. Physical demands in elite team handball: Comparisons between male and female players. J Sports Med Phys Fitness. 2015;55(9):878–91.
3. Engebretsen L, Soligard T, Steffen K, Alonso JM, Aubry M, Budgett R, et al. Sports injuries and illnesses during the London Summer Olympic Games 2012. Br J Sports Med. 2013;47(7):407–14.
4. Michalsik LB. On-court physical demands and physiological aspects in elite team handball. Handball sports medicine: Basic science, injury management and return to sport. 2018;15–33.
5. Ament W, Verkerke GJ. Exercise and fatigue. Sports med. 2009;39:389–422.

6. Gandevia SC, Allen GM, Butler JE, Taylor JL. Supraspinal factors in human muscle fatigue: evidence for suboptimal output from the motor cortex. J Physiol. 1996;490(2):529–36.

7. Westerblad H, Lee JA, Lannergren J, Allen DG. Cellular mechanisms of fatigue in skeletal muscle. Am J Physiol Cell Physiol. 1991;261(2):C195–209.

8. Thorlund JB, Michalsik LB, Madsen K, Aagaard P. Acute fatigue-induced changes in muscle mechanical properties and neuromuscular activity in elite handball players following a handball match. Scand J Med Sci Sports. 2008;18(4):462–72.

9. Póvoas SCA, Ascensão AAMR, Magalhães J, Seabra AFT, Krustrup P, Soares JMC, et al. Analysis of fatigue development during elite male handball matches. J Strength Cond Res. 2014;28(9):2640–8.

10. Bedo BLS, Pereira DR, Moraes R, Kalva-Filho CA, Will-de-Lemos T, Santiago PRP. The rapid recovery of vertical force propulsion production and postural sway after a specific fatigue protocol in female handball athletes. Gait Posture. 2020;77:52–8.

11. Thorlund JB, Michalsik LB, Madsen K, Aagaard P. Acute fatigue-induced changes in muscle mechanical properties and neuromuscular activity in elite handball players following a handball match. Scand J Med Sci Sports. 2008;18(4):462–72.

12. Langevoort G, Myklebust G, Dvorak J, Junge A. Handball injuries during major international tournaments. Scand J Med Sci Sports. 2007;17(4):400–7.

13. Martín-Guzón I, Muñoz A, Lorenzo-Calvo J, Muriarte D, Marquina M, De la Rubia A. Injury prevalence of the lower limbs in handball players: A systematic review. Int J Environ Res Public Health. 2021;19(1):332.

14. Edouard P, Chatard JC, Fourchet F, Collado H, Degache F, Leclair A, et al. Invertor and evertor strength in track and field athletes with functional ankle instability. Isokinet Exerc Sci. 2011;19(2):91–6.

15. Ersoz M, Atalay NS, Kumbara F, Akyuz M. Investigation of effect of age, gender and limb dominance on ankle evertor/invertor peak torque ratios of healthy volunteers. J Phys Ther Sci. 2009;21(3):263–7.

16. Aagaard P, Simonsen EB, Trolle M, Bangsbo J, Klausen K. Isokinetic hamstring/quadriceps strength ratio: influence from joint angular velocity, gravity correction and contraction mode. Acta Physiol Scand. 1995;154(4):421–7.

17. Sangnier S, Tourny-Chollet C. Comparison of the decrease in strength between hamstrings and quadriceps during isokinetic fatigue testing in semiprofessional soccer players. Int J Sports Med. 2007;28(11):952–7.

18. van Melick N, van der Weegen W, van der Horst N. Quadriceps and hamstrings strength reference values for athletes with and without anterior cruciate ligament reconstruction who play popular pivoting sports, including soccer, basketball, and handball: a scoping review. J Orthop Sports Phys Ther. 2022;52(3):142–55.

19. Pontaga I. Ankle joint evertor–invertor muscle torque ratio decrease due to recurrent lateral ligament sprains. Clin Biomech. 2004;19(7):760–2.

20. Cozette M, Leprêtre PM, Doyle C, Weissland T. Isokinetic Strength Ratios: Conventional Methods, Current Limits and Perspectives. Front Physiol. 2019;10.

21. Gleeson NP, Mercer TH. The utility of isokinetic dynamometry in the assessment of human muscle function. Sports med. 1996;21:18–34.

22. Faul F, Erdfelder E, Lang AG, Buchner A. G*Power 3: a flexible statistical power analysis program for the social, behavioral, and biomedical sciences. Behav Res Methods. 2007 May;39(2):175–91.

23. Andrade MS, de Carvalho Koffes F, Benedito-Silva AA, da Silva AC, de Lira CAB. Effect of fatigue caused by a simulated handball game on ball throwing velocity, shoulder muscle strength and balance ratio: a prospective study. BMC Sports Sci Med Rehabil. 2016;8:1–7.

24. Esmaeili H, Sharifi A. Bilateral asymmetry of hamstring to quadriceps isokinetic torque ratio in different angular velocities in professional basketball players. Knee. 2022 Aug 1;37:3–9.

25. Aydoğ E, Aydoğ ST, Çakci A, Doral MN. Reliability of isokinetic ankle inversion-and eversion-strength measurement in neutral foot position, using the Biodex dynamometer. Knee Surg Sports Traumatol Arthrosc. 2004;12:478–81.

26. Fong DTP, Ha SCW, Mok KM, Chan CWL, Chan KM. Kinematics analysis of ankle inversion ligamentous sprain injuries in sports: five cases from televised tennis competitions. Am J Sports Med. 2012;40(11):2627–32.

27. Ameer MA. Kinematic comparison between single and double-leg jump landings in sagittal plane for male hand ball players. Turk J Sport Exer. 2016;18(3):108–15.

28. Borg G. Borg's perceived exertion and pain scales. Hum kinet; 1998.

29. Bove AM, Lynch AD, DePaul SM, Terhorst L, Irrgang JJ, Fitzgerald GK. Test-retest reliability of rating of perceived exertion and agreement with 1-repetition maximum in adults. J Orthop Sports Phys Ther. 2016;46(9):768–74.

30. Scott TJ, Black CR, Quinn J, Coutts AJ. Validity and reliability of the session-RPE method for quantifying training in Australian football: a comparison of the CR10 and CR100 scales. J Strength Cond Res. 2013;27(1):270–6.

31. Cohen J. Statistical power analysis for the behavioral sciences. routledge; 2013.

32. Pinto MD, Blazevich AJ, Andersen LL, Mil-Homens P, Pinto RS. Hamstring-to-quadriceps fatigue ratio offers new and different muscle function information than the conventional non-fatigued ratio. Scand J Med Sci Sports. 2018;28(1):282–93.

33. Massamba A, Hucteau E, Mallard J, Ducrocq GP, Favret F, Hureau TJ. Exercise-Induced Fatigue in Hamstring versus Quadriceps Muscles and Consequences on the Torque–Duration Relationship in Men. Med Sci Sports Exerc. 2022;54(12):2099–108.

34. Greco CC, Da Silva WL, Camarda SRA, Denadai BS. Fatigue and rapid hamstring/quadriceps force capacity in professional soccer players. Clin Physiol Funct Imaging. 2013;33(1):18–23.

35. Greig M, Siegler JC. Soccer-specific fatigue and eccentric hamstrings muscle strength. J Athl Train. 2009;44(2):180–4.

36. Delextrat A, Baker J, Cohen DD, Clarke N. Effect of a simulated soccer match on the functional hamstrings-to-quadriceps ratio in amateur female players. Scand J Med Sci Sports. 2013;23(4):478–86.

37. Greig M. The influence of soccer-specific fatigue on peak isokinetic torque production of the knee flexors and extensors. Am J Sports Med. 2008;36(7):1403–9.

38. Behrens M, Mau-Moeller A, Wassermann F, Bruhn S. Effect of fatigue on hamstring reflex responses and posterior-anterior tibial translation in men and women. PLoS One. 2013;8(2):e56988.

39. Nocella M, Colombini B, Benelli G, Cecchi G, Bagni MA, Bruton J. Force decline during fatigue is due to both a decrease in the force per individual cross-bridge and the number of cross-bridges. J Physiol. 2011;589(13):3371–81.

40. Gandevia SC. Spinal and supraspinal factors in human muscle fatigue. Physiol Rev. 2001;81(4):1725–89.

41. Fitts RH. Cellular mechanisms of muscle fatigue. Physiol Rev. 1994;74(1):49–94.

42. de Oliveira Pires F. Thomas Kuhn's 'Structure of Scientific Revolutions' applied to exercise science paradigm shifts: example including the Central Governor Model. Br J Sports Med. 2013;47(11):721–2.

43. Noakes TD. Physiological models to understand exercise fatigue and the adaptations that predict or enhance athletic performance. Scand J Med Sci Sports. 2000;10(3):123–45.

44. Marshall PWM, Lovell R, Knox MF, Brennan SL, Siegler JC. Hamstring fatigue and muscle activation changes during six sets of Nordic hamstring exercise in amateur soccer players. J Strength Cond Res. 2015;29(11):3124–33.

45. Bachasson D, Decorte N, Wuyam B, Millet GY, Verges S. Central and peripheral quadriceps fatigue in young and middle-aged untrained and endurance-trained men: A comparative study. Exp Biol Med. 2016;241(16):1844–52.

46. Pinto MD, Blazevich AJ, Andersen LL, Mil-Homens P, Pinto RS. Hamstring-to-quadriceps fatigue ratio offers new and different muscle function information than the conventional non-fatigued ratio. Scand J Med Sci Sports. 2018;28(1):282–93.

47. Chen TC, Lin KY, Chen HL, Lin MJ, Nosaka K. Comparison in eccentric exercise-induced muscle damage among four limb muscles. Eur J Appl Physiol. 2011;111:211–23.

48. Hamada T, Sale DG, MacDougall JD, Tarnopolsky MA. Interaction of fibre type, potentiation and fatigue in human knee extensor muscles. Acta Physiol Scand. 2003;178(2):165–73.

49. Smeets A, Malfait B, Dingenen B, Robinson MA, Vanrenterghem J, Peers K, et al. Is knee neuromuscular activity related to anterior cruciate ligament injury risk? A pilot study. Knee. 2019;26(1):40–51.

50. Akaras E, Güzel NA, Çobanoğlu G, Ecemiş ZB, Şimşek BÜ. The effect of hamstring/quadriceps muscle strength ratio on plantar pressure distribution-A pilot study. Turk J of Kin. 2024;10(4):298–305.

51. Nagel A, Fernholz F, Kibele C, Rosenbaum D. Long distance running increases plantar pressures beneath the metatarsal heads: A barefoot walking investigation of 200 marathon runners. Gait Posture. 2008;27(1):152–5.

52. Sangnier S, Tourny-Chollet C. Comparison of the decrease in strength between hamstrings and quadriceps during isokinetic fatigue testing in semiprofessional soccer players. Int J Sports Med. 2007;28(11):952–7.

53. Lin YH, Li CW, Tsai LY, Liing RJ. The effects of muscle fatigue and proprioceptive deficits on the passive joint senses of ankle inversion and eversion. Isokinet Exerc Sci. 2008;16(2):101–5.

54. Rodrigues KA, Soares RJ, Tomazini JE. The influence of fatigue in evertor muscles during lateral ankle sprain. The Foot. 2019;40:98–104.

55. Silva BARS, Martinez FG, Pacheco AM, Pacheco I. Effects of the exercise-induced muscular fatigue on the time of muscular reaction of the fibularis in healthy individuals. Rev Bras Med Esporte. 2006;12:85–9.

56. Sandrey MA, Kent TE. The effects of eversion fatigue on frontal plane joint position sense in the ankle. J Sport Rehabil. 2008;17(3):257–68.

57. Castillo GB, Brech GC, Luna NMS, Tarallo FB, Soares-Junior JM, Baracat EC, et al. Influence of invertor and evertor muscle fatigue on functional jump tests and postural control: A prospective cross-sectional study. Clinics. 2022;77:100011.

58. Kaminski TW, Hartsell HD. Factors contributing to chronic ankle instability: a strength perspective. J Athl Train. 2002;37(4):394.

59. Calmels PM, Nellen M, van der Borne I, Jourdin P, Minaire P. Concentric and eccentric isokinetic assessment of flexorextensor torque ratios at the hip, knee, and ankle in a sample population of healthy subjects. Arch Phys Med Rehabil. 1997;78(11):1224–30.

Revised Manuscript with Track Changes

Effect of simulated handball match-induced fatigue on isokinetic hamstring-to-quadriceps ratio and evertor-to-invertor ratio in professional players

Ali Shirani Dastjerdi[1], Hamed Esmaeili[1*], Morteza Sadeghi[1], Behzad Bashiri[2]

[1] Department of Sport Injuries and Corrective Exercises, Faculty of Sport Sciences, University of Isfahan, Isfahan, Iran

[2] Department of Biomedical Engineering, Faculty of Engineering, International University of Science and Technology in Kuwait, Ardiya, Kuwait

Short Title: Effect of simulated handball match-induced fatigue on lower limb peak torques

* Corresponding author: Hamed Esmaeili

Email: H.esmaeili@spr.ui.ac.ir

Abstract

Knee and ankle injuries are the most common injuries in handball players. Many of these injuries occur toward the end of the game when fatigue sets in. The underlying mechanisms of this phenomenon are not well understood. This study investigated the effect of a handball match-simulation protocol on the hamstring-to-quadriceps (H:Q) and evertor-to-invertor (E:I) peak torque ratios. Thirty professional male handball players from Iranian super league teams participated in this study. Isokinetic concentric peak torque of the hamstrings, quadriceps, ankle invertor, and ankle evertors were measured at angular velocities of 60, 120, and 180 deg/s) before and after the simulated handball match-induced fatigue protocol using a Biodex Isokinetic Dynamometer. Peak torque ratio of H:Q and E:I were calculated and compared between pre- and post-fatigue conditions. Fatigue significantly reduced H:Q peak torque ratio at 60 (p=0.049), 120 (p=0.002) and 180 (p=0.014) deg/s, as well as E:I peak torque ratio at 60 (p=0.010), 120 (p=0.003) and 180 (p=0.003) deg/s. These changes could contribute to an increased risk of anterior cruciate ligament (ACL) tears and lateral ankle sprains. Given the greater reduction in the H:Q peak torque ratio in hamstring muscles, targeted strengthening and improving the endurance of these muscles is recommended for professional handball players to mitigate fatigue effects.

Keywords: Isokinetic, Knee, Ankle, Fatigue, Handball, Hamstring-to-quadriceps ratio, Torque, Injury risk

Introduction

Handball, as a complex and highly demanding intermittent sport, involves multiple high-intensity runs, frequent body contact, and other high-intensity actions to overcome opponents (1). A handball match involves a large number of repeated accelerations, sprints, jumps, blocking, pushing and rapid changes in moving directions (2). Sudden changes of direction, decelerations and accelerations, vertical and horizontal jumps, throws, thrusts, pulls, and powerful, fast-paced clashes are among the movements of handball sport that have made it a sport with high risk of injury (3).

These physical demands are expected to lead to fatigue, which could be at least partially responsible for the reduction in the quantity and quality of high-intensity actions toward the end of a match compared to the beginning (4). Fatigue can be categorized into two types: central fatigue, which refers to a decline in performance due to factors occurring near the neuromuscular synapses, and peripheral fatigue, characterized by a reduction in muscle contraction strength caused by processes further from these synapses (5–7). Both forms of fatigue can appear during training sessions or matches, potentially leading to impaired coordination, muscle mechanical properties and neuromuscular activity, and a higher frequency of mistakes (8). During elite male handball matches, there was a decrease in high-intensity running time, demanding actions, and time spent with a heart rate above 80% of maximum from the first half to the second (9). Metabolic markers such as plasma free fatty acids, glycerol, glucose, and uric acid increased during the first half, with further increases in free fatty acids and glycerol during the second half (9). These changes led to decline in sprint performance and reduction in vertival jump height compared to baseline levels, indicating significant fatigue development (9). Moreover, handball match-induced fatigue has been shown to increase center of

pressure area during landing and decrease maximum propulsion force for countermovement jump (10).

It has been shown that players who play 70 percent of the total match time experience changes such as reduction in high-intensity running, reduction in heart rate, reduction in technical and impaired physical performance, resulting from fatigue (11). This condition could increase demand on musculoskeletal system and leaves players at high risk of injury. Notably, the highest number of injuries during six periods of international competitions occurred between minutes 16 to 30 of the first half and 46 to 60 of the second half (12).

Given these findings, it can be suggested that physical fatigue deteriorates neuromuscular and sensorimotor control, which may elevate the risk of injury. Handball players report a higher prevalence of lower limb injuries than injuries to the upper limbs, neck, or head (12,13). For instance, approximately 30% of injuries occur in the knee, and 25% in the ankle (13).

The hamstring-to-quadriceps (H:Q) peak torque ratio in the knee joint and evertor to invertor (E:I) peak torque ratio in the ankle are two risk factors used to assess muscle balance or imbalance in the knee and ankle (14–17). Deterioration in these ratios can increase the risk of injury. A decreased H:Q ratio implies that the quadriceps are disproportionately stronger than the hamstrings, which may stress the anterior cruciate ligament (ACL) and may lead to injuries (18). A decrease in the E:I peak torque ratio is linked to a higher risk of lateral ankle sprains (19).

Isokinetic muscle peak torque is a critical metric in assessing injury risk, particularly in sports and rehabilitation settings. Isokinetic testing can identify neuromuscular deficits, such as reduced torque production or delayed muscle activation, which are often linked to injury risk (20,21). For example, a decline in hamstring peak torque

has been associated with an increased risk of hamstring strains in soccer players (21).

Given the high incidence of lower limb injuries among handball players, particularly when players are fatigued during matches, it is crucial to investigate the effects of match-induced fatigue on H:Q and E:I peak torque ratios in handball. The aim of this study was to evaluate the effects of simulated handball match-induced fatigue on the isokinetic hamstring-to-quadriceps and invertor-to-evertor ratios in professional handball players.

Materials and Methods

Participants

Thirty professional male handball players from Iranian Super League teams (age: 27 ± 3.1 years; height: 186.9 ± 7.3 cm; mass: 86.8 ± 6.7 kg) participated in this study. A prior power analysis revealed that for a statistical power of 0.9 at an alpha level of 0.05 and an effect size of 0.41, a sample size of at least 26 participants is required (22). The effect size was chosen from a previous study that had used a similar fatigue protocol (23). Participant recruitment for this study began on August 15, 2023, and concluded on November 30, 2023. The inclusion criteria for participation required all subjects to have engaged in competitive handball for a minimum of 5 years and to participate in more than thee training sessions per week. During the preseason, the players completed 8–10 training sessions per week, while during the season, they attended 5–7 sessions per week. All participants were active team members and participated in every match. Volunteers who reported injuries in the lower limbs in the last 6 months were excluded. All participants provided written informed consent. This study was approved by the University of Isfahan Ethics Committee and conducted in accordance with the Declaration of Helsinki.

Testing preparation

Initially, participants were briefed on the testing procedure. The participants began with an 8-minute warmup on a stationary bicycle with no resistance at moderate velocity (70-80 revolutions per minute) (24). Next, they sat on the dynamometer chair (Biodex Multi-joint System 3, Biodex Medical Systems, USA) which was adjusted so they had free and full movement in knee and ankle joints. Participants were fixed with two horizontal and vertical belts, and the test limb was fixed to the arm of the device. The seat of the device was adjusted to an angle of 100 degrees. The arm of the device was adjusted to a tilit angle of 0 deg. The movement axis of the knee was aligned parallel to the movement axis of the device. For the ankle test, the participant's ankle was positioned on the footplate and the foot was secured using two Velcro straps.

For each test session, the range of motion of ankle and knee joints was determined first (25). Concentric isokinetic peak torque for quadriceps, hamstring, ankle invertors, and ankle evertors was measured at three different angular velocities of 60, 120 and 180 deg/s by a Biodex Multi-joint System 3 dynamometer (Biodex Medical Systems, USA) on the landing leg in a random order. The landing leg was defined as the leg that players use to land after jumping (26,27). Each angular velocity was measured with one set of six repetitions. In each test, participants were instructed to perform the movements with maximum effort. Verbal encouragement was provided during the test. Peak torques were assessed at baseline and immediately after the handball-specific fatigue protocol to examine its effects on the outcomes.

Fatiguing protocol

A previously used fatigue protocol was utilized to simulate match-induced fatigue (10) which included special movements during a handball match, such as sprinting, jumping, landing, sidestep cutting, and lateral and backward shifting (Fig 1, caption for more details). The protocol was divided into gradually increasing rounds, requiring participants to complete each round as quickly as possible. The number of rounds corresponded to the number of laps in the circuit (e.g., round I: 1 lap; round II: 2 laps; and so on). For each round, time of effort, heart rate using Polar F4 Blue ICE, and the rate of perceived exertion (RPE) using the 15-point Borg scale with a a range of 6-20 were recorded (28). In this Borg Scale, a score of six indicates no fatigue, while scores between 17 and 20 indicate the inability to continue the exercises. During the trials, if participants reported a score of 17 or higher at the end of a round, the fatigue protocol ended. If they reported a lower score, they performed another round until they reached a score of 17. Borg scale has been shown to have high test-retest reliability (29,30).

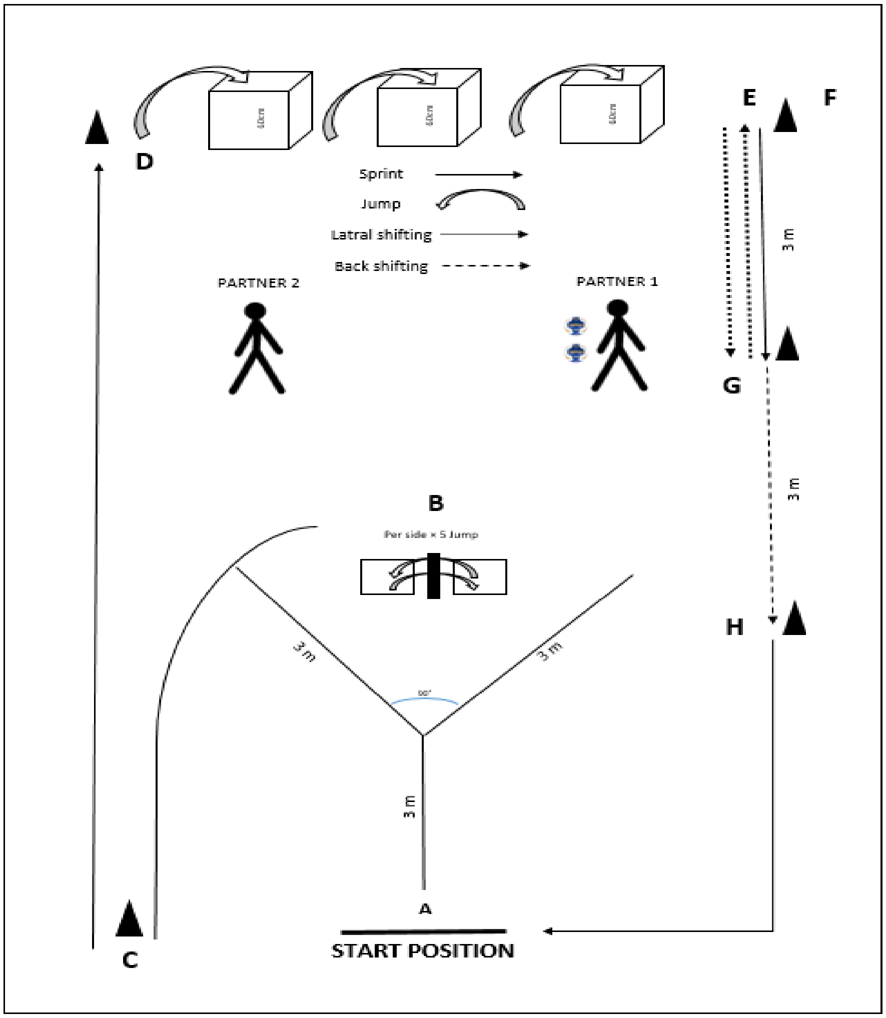

Figure 1. Illustration of the fatigue protocol based on specific movements performed by handball athletes. The athletes performed the following actions in all of the stations as fast as possible: (A) Acceleration and sidestep cutting maneuver for the right and run until the end of the 3-m distance; receive a pass (at the end of the 3-m length) from a partner positioned 2.5 m away (Partner 1); backshift until the 90°; run 3 m for the left side, and perform an accurate throw to a partner positioned 2.5 m away (Partner 2); this task was performed twice per lap; (B) Ten double-leg lateral jumping/landing over a barrier (20-cm high), performed as fast as possible; (C) Sprint; (D) Double-leg plyometric jumps, performed as fast as possible; (E) Lateral shifting; (F) Sprint; (G) Back Shifting; and (H) Sprint with a 90° change of direction. The protocol was divided into rounds, where the round number represents the number of laps in the circuit (e.g., round I: 1 lap, round II: 2 laps, round III: 3 laps, and so on) until the participant is unable to continue performing the protocol.

Data analysis

Peak knee flexion, knee extension, ankle inversion and ankle eversion torque were recorded for each repetition. Between each set of the test, participants rested for 2-

min. To calculate the H:Q ratio, peak flexion torque was divided by the peak extensor torque [15 Bilateral]. To calculate the E:I ratio, peak eversion torque was divided by the peak inversion torque (19). Peak torques per body mass, along with the peak torque ratios of H:Q and E:I, were used in subsequent analyses.

Statistical Analysis

Shapiro-Wilk test was used to ensure normality of data distribution. To investigate the effect of fatigue on the outcomes, a 2 (condition: pre- and post-fatigue) × 3 (angular velocity: 60, 120, and 180 deg/s) repeated measures ANOVA was used. Holm-Bonferroni adjustment was used for multiple comparisons. Effect size (ES), reported using partial eta squared, η_p^2, was classified as small (η_p^2 = 0.01), medium (η_p^2 = 0.06), and large (η_p^2 = 0.14) (31). All statistical analyses were performed at a significance level of $p < 0.05$ using SPSS software, version 18.0 (SPSS Inc, Chicago IL, USA).

Results

Fatigue Markers

Each participant executed a particular number of laps to achieve fatigue, ranging between four and eleven (5.8 ± 2.4 laps or 276 ± 79.2 s). Significant increases were observed for heart rate ($F_{(1,29)} = 341.58$; $p < 0.001$) and RPE ($F_{(1,29)} = 392.42$; $p < 0.001$) (table 1).

Table 1. Mean and standard deviation (±) of the fatigue parameters.

	Baseline	Fatigue State	F	P
**Heart rate**	64.26 ± 8.2	186.31 ± 7.4	341.58	**< 0.001**
**RPE**	6 ± 0	20 ± 0	392.42	**< 0.001**

Bold values indicate significant difference in p ≤ 0.05.

Effect of fatigue on peak torques

The results showed that the main effect of fatigue ($F_{(1,29)} = 36.14$, $p < 0.01$, ES = 0.56) and the main effect of angular velocity ($F_{(2,58)} = 87.58$, $p < 0.01$, ES = 0.751) were significant, but the interaction effect was not significant ($F_{(2,58)} = 2.20$, $p = 0.120$, ES = 0.070) on the hamstring peak torque to body mass ratio. Multiple comparisons showed that fatigue decreased hamstring peak torque in all angular velocities (Table 2).

For the quadriceps peak torque to body mass ratio, the results showed significant main effects for fatigue ($F_{(1,29)} = 7.18$, $p = 0.012$, ES = 0.198) and angular velocity ($F_{(2,58)} = 296.68$, $p = 0.001$, ES = 0.911), and a significant fatigue × angular velocity interaction effect ($F_{(2,58)} = 4.20$, $p = 0.020$, ES = 0.127). This indicates that fatigue affects quadriceps peak torque differently at various angular velocities. Post-hoc tests revealed that fatigue was associated with a decrease in quadriceps peak torque at 60 deg/s and 180 deg/s, but not at 120 deg/s (Table 2).

Table 2. Comparison of hamstring and quadriceps peak torque ratio (in Nm/body mass) in the pre- and post-fatigue at different angular velocities. Data are presented in Mean ± SD.

		Pre	Post	Mean Difference	Standard Error	95% Confidence Interval	t	p_{holm}
**Hamstring**	60 deg/s	1.83 ± .36	1.56 ± .25	0.27333	0.0649	(0.476 - 1.330)	4.214	**0.001**
	120 deg/s	1.55 ± .24	1.43 ± .26	0.12247	0.0360	(0.280 - 1.077)	3.398	**0.006**
	180 deg/s	1.32 ± .23	1.18 ± .23	0.13907	0.0198	(0.823 - 1.808)	7.031	**0.001**
**Quadriceps**	60 deg/s	2.84 ± .35	2.65 ± .43	0.1827	0.0649	(0.186 - 0.961)	2.81	**0.026**
	120 deg/s	2.33 ± .34	2.24 ± .31	0.0852	0.0564	(-0.092 - 0.638)	1.51	0.141
	180 deg/s	1.68 ± .25	1.60 ± .27	0.9749	0.0668	(0.036 - 0.772)	2.19	**0.041**

Bold values indicate significant difference in $p \leq 0.05$.

Regarding the invertor peak torque to body mass ratio, the results of ANOVA showed a significant main effect of fatigue ($F_{(1,29)} = 12.97$, $p = 0.001$, ES = 0.309) and angular velocity ($F_{(2,58)} = 71.46$, $p = 0.001$, ES = 0.711), but no significant fatigue × angular velocity interaction ($F_{(2,58)} = 2.11$, $p = 0.130$, ES = 0.068). Multiple comparisons showed that, after fatigue, invertor peak torque decreased only in 60 deg/s (Table 3).

Repeated measures analysis of variance showed significant main effects of fatigue ($F_{(1,29)}$ = 44.01, p = 0.001, ES = 0.603) and angular velocity ($F_{(2,58)}$ = 99.48, p = 0.001, ES = 0.774), and a significant fatigue × angular velocity interaction ($F_{(2,58)}$ = 3.35, p = 0.042, ES = 0.104) on the evertor peak torque to body mass ratio. The handball-specific fatigue protocol was associated with decrease in evertor peak torque in all three angular velocities (Table 3).

Table 3. Comparison of invertor and evertor peak torque ratio (in Nm/body mass) in the pre- and post-fatigue at different angular velocities. Data are presented in Mean ± SD.

		Pre	Post	Mean Difference	Standard Error	95% Confidence Interval	t	p_{holm}
**Invertor**	60 deg/s	0.44 ± .10	0.39 ± .10	0.0469	.01233	(0.290 - 1089)	3.80	**.003**
	120 deg/s	0.37 ± .08	0.35 ± .09	0.0240	.01194	(-0.006 - 0.734)	2.01	.161
	180 deg/s	0.31 ± .09	0.30 ± .06	0.0165	.01179	(-0.111 – 0.617)	1.40	.345
**Evertor**	60 deg/s	0.42 ± .12	0.33 ± .08	0.08779	.01389	(0.684 – 1.612)	6.319	**.001**
	120 deg/s	0.35 ± .09	0.27 ± .06	0.07345	.01292	(0.586 – 1.478)	5.685	**.001**
	180 deg/s	0.27 ± .08	0.21 ± .05	0.05667	.01202	(0.435 – 1.276)	4.714	**.001**

Bold values indicate significant difference in p ≤ 0.05.

Effect of fatigue on peak torque ratios

ANOVA test showed significant main effects of fatigue ($F_{(1,29)}$ = 18.143, p = 0.001, ES = 0.385) and angular velocity ($F_{(2,58)}$ = 60.019, p = 0.001, ES = 0.674), but no significant fatigue × angular velocity interaction effect ($F_{(2,58)}$ = 0.526, p = 0.594, ES = 0.018) on H:Q peak torque ratio. Fig 2 illustrates H:Q peak torque ratio in the pre- and post-fatigue in different angular velocities. As shown, fatigue decreased H:Q ratio in 60 ($t_{(1,29)}$ = 2.662, p = 0.049), 120 ($t_{(1,29)}$ = 4.202, p = 0.002) and 180 ($t_{(1,29)}$ = 3.327, p = 0.014) deg/s.

ANOVA test showed significant main effects for fatigue ($F_{(1,29)}$ = 17.39, p = 0.001, ES = 0.375) and angular velocity ($F_{(2,58)}$ = 29.90, p = 0.001, ES = 0.508), but no significant fatigue × angular velocity interaction effect ($F_{(2,58)}$ = 1.63, p = 0.206, ES = 0.053) on E:I. As shown in Fig 3, fatigue decreased E:I in 60 ($t_{(1,29)}$ = 3.469, p = 0.010), 120 ($t_{(1,29)}$ = 4.164, p = 0.003) and 180 ($t_{(1,29)}$ = 4.198, p = 0.003) deg/s.

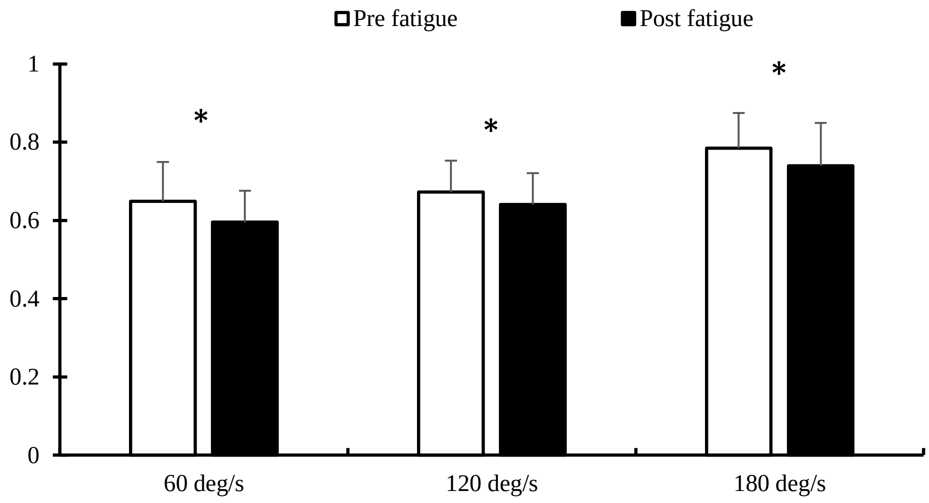

Fig 2. Comparison of H:Q peak torque ratio at different angular velocities in pre- and post-fatigue. (*) stands for significant difference in $p \leq 0.05$.

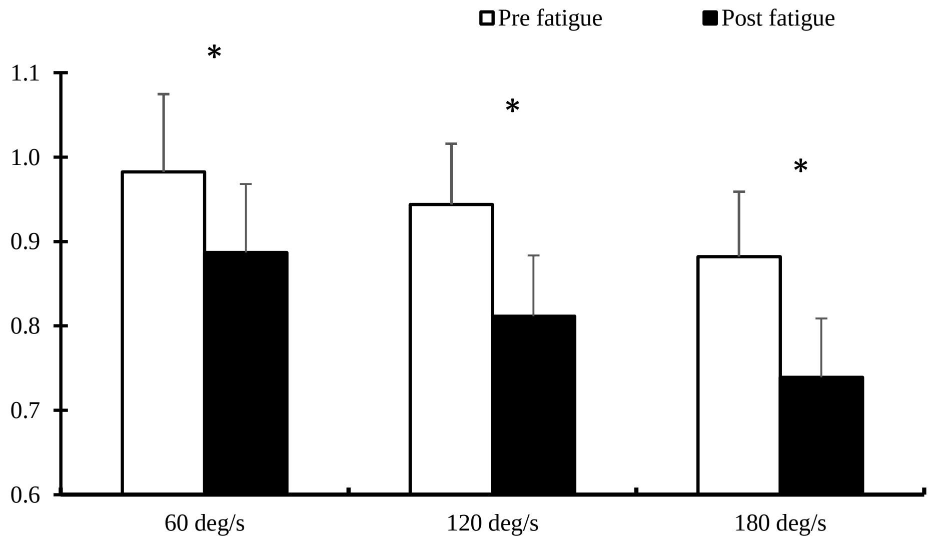

Fig 3. Comparison of E:I peak torque ratio in different angular velocities in pre-and-post fatigue. (*) stands for significant difference in $p \leq 0.05$.

Discussion

Knee and ankle injuries are the most common injury sites among handball players, especially when players experience fatigue during matches. The effect of match-

induced fatigue on hamstring, quadriceps, ankle invertors and evertors, and their subsequent ratios is not well understood. The aim of the current study was to investigate the effect of simulated handball match-induced fatigue on H:Q and E:I peak torque ratios in Iranian handball Super League players. Our results showed that fatigue decreased peak hamstring and quadriceps muscles torque per body mass and the H:Q peak torque ratio. Also, the results showed that fatigue was associated with a decrease in peak invertor and evertor muscles torque per body mass and decrease in E:I peak torque ratio. In line with our results, Pinto *et al*. and Sangnier and Tourny-Chollet showed decreases in knee flexor and extensor torque and hamstring-to-quadriceps ratios (17,32). Pinto et al showed that after completing a 30-repetition isokinetic fatigue test protocol in professional soccer players, the knee extensor and flexor moments decreased and the H:Q was decreased at the end of the test due to a significant reduction in knee flexor moment as well (32). Sangnier and Tourny-Chollet also using an isokinetic fatigue protocol on professional soccer players showed decreased strength of hamstring and quadriceps muscles (17). Massamba *et al*. showed that after a 5-min all-out exercise training-induced fatigue, the maximum voluntary isometric force of quadriceps and hamstring muscles of healthy men decreased (33). Our observed decreases in hamstring and quadriceps muscle peak torques in the current study, indicate effect of a simulated handball match-induced fatigue on knee flexor and extensor muscles strength and force output. While it has been well known that fatigue affects peak force output, training modalities seem to have different effects on force output reduction (34–38). After completion of different fatigue protocols, similar reductions in force output are observed in knee flexor and extensor muscles concentric or isometric measurements (33,35). The decrease in strength and torque output may result from a reduced ability of the muscles to generate force, which is associated with a decrease in the number of cross-bridges generating force and the number of cross-bridges actively working in

the muscle (39). This reduction can result from either general or localized muscle fatigue. Fatigue has both central and peripheral origins (40). Peripheral fatigue is characterized by inability of the muscle to contract effectively (41). Central fatigue, on the other hand, involves a significant reduction in the neural drive to the muscles (40), typically occuring after prolonged efforts. Therefore, it is plausible that the fatigue induced by our protocol has peripheral origins. However, according to the central governor model (42), the central component may also play a role in this type of exercise. This model suggests that the central nervous system interacts with the muscles to protect vital organs (e.g., the heart and brain) by reducing neural drive in high-demand situations, even during short-term exercises (43). Thus, the fatigue protocol used in the present study may induce both peripheral and central fatigue.

The proposed fatigue protocol was designed to induce high levels of physiological and psychological stress, ultimately leading to exhaustion. This was demonstrated by the heart rate (HR) recorded at the point of exhaustion, which reached 186.31 ± 7.4 bpm, approximately 96% of the predicted maximum HR. Additionally, participants reported maximum values on the RPE score of 20, further confirming the extreme cardiologic stress induced by the protocol. These findings, along with the observed reductions in quadriceps, hamstring invertor and evertor muscles peak torque per body mass and their respective ratios, strongly suggest that the protocol effectively induces fatigue. These results indicate that this protocol reliably simulates fatigue in handball players, making it a valuable tool for both training and research purposes.

Our results showed that knee flexor muscles are influenced by handball match induced fatigue as well as knee extensors. Changes in fatigued condition are repetitively reported for knee extensor muscles (44,45). However, our results are inconsistent with those of Marshal *et al.* who showed soccer simulation match-

induced fatigue had no effect on hamstring muscles peak torque (44). This inconsistency could be attributed to differences in the population and fatigue protocol. Marshal *et al.* studied soccer players and a soccer simulation match, whereas the current study included professional handball players and used a handball match-simulating protocol which engaged both hamstring and quadriceps muscles.

Our results showed that H:Q peak torque ratio decreased after fatigue protocol. The reduction in this ratio was associated with a decrease in hamstring and quadriceps muscles peak torques. Since this ratio is calculated by dividing hamstring peak torque by quadriceps peak torque, it seems that hamstring muscles are more affected by fatigue. Pinto *et al.* showed that after a fatigue protocol, hamstring muscles torque decreased to a greater extent than that of quadriceps (46), which is in line with the results of our study. Hamstring muscles besides their complex muscular architecture and multi-joint function, comprise a significant number of type II fibers and are less utilized in most daily activities, making them more susceptible to muscle damage and fatigue (47,48). Although the mechanism of hamstring and ACL injuries is still a subject of debate, it is well-established that neuromuscular fatigue is an important risk factor (49). For example, changes in hamstring muscle coordination, alterations in hamstring length during late swing phase of running cycle, and altered ACL-hamstring reflex are associated with muscle stiffness regulation and knee stability after a fatigue protocol (32). Additionally, it has been shown that a decreased H:Q is associated with increased loading on the forefoot and toe regions (50). The increased loading could lead to increased risk of stress fracture in the forefoot region (51). Overall, these findings also indicate that knee flexors should be well-trained to enhance their force production capacity and endurance, thus reducing susceptibility to hamstring and ACL injuries. Additionally, a reduction in the H:Q peak torque ratio increases the risk of ACL injury (52).

The results of the present study demonstrated that following a simulated handball game fatigue protocol, invertor and evertor muscles peak torque decreased. Additionally, a decrease in E:I muscles peak torque ratio was observed in the post-fatigue condition. The reduction in isokinetic maximum torque of the invertor and evertor muscles can be attributed to the effects of the fatigue protocol, as previously mentioned. These changes in muscle torques have implications for foot and ankle function. Lin and colleagues, using an isokinetic concentric-eccentric fatigue protocol, investigated the effects of invertor and evertor muscles fatigue and found that it leads to a decrease in ankle joint position sense in the frontal plane (53). Similarly, Rodriguez et al. demonstrated that after inducing fatigue by maintained eversion and plantar flexion at 70% of the maximum voluntary isometric contraction until a 10% decline in force, neuromuscular control was compromised in fatigued evertor muscles (54). Bars Silva *et al.* induced fatigue through active localized ankle eversion exercises against resistance and demonstrated that exercise-induced muscle fatigue significantly increases the reaction time of the peroneal muscles (55). This delayed reaction time can impair the muscles' ability to respond quickly to sudden changes in ankle position, potentially increasing the risk of ankle injuries (55). Sandrey and Kent used a fatigue protocol targeting the ankle evertors, followed by tests to measure their ability to detect and replicate specific ankle positions in the frontal plane (56). They also assessed absolute error in joint position sense, as an indicator of proprioceptive depth perception; they demonstrated that eversion fatigue negatively affects the ankle's ability to sense its position in the frontal plane (56). Castillo et al. found that fatigue in the invertor and evertor muscles significantly impaired performance in functional jump tests (57). They reported that participants experienced reduced jump height, distance, and control after muscle fatigue, indicating a decline in dynamic stability and power output (57). Thus, fatigue-

induced reductions in evertor muscle peak torque can decrease proprioceptive sensation and increase the risk of ankle sprains

Our results indicate that fatigue led to a decrease in the E:I peak torque ratio. This decrease in the E:I peak torque ratio is associated with a higher risk of lateral ankle sprains (19). As shown, the decline in evertor muscle torque had greater contribution to decrease in E:I peak torque ratio than that of invertor muscles. It has been shown that prior to the occurrence of lateral ankle sprains, the E:I torque ratio decreases (19,58). In our study, fatigue was associated with decreases in evertor muscles peak torque at 60, 120, and 180 deg/s . However, the effect of fatigue on invertor muscles was different. Specifically, fatigue only decreased invertor muscle peak torque at 60 deg/s, with no effect at 120 and 180 deg/s. Based on these results, it seems that fatigue induced by a handball simulation protocol could contribute to an increased risk of lateral ankle sprains. The E:I peak torque ratio is derived from dividing the peak torque of the evertor muscles by that of the invertor muscles. Given that the reduction in evertor muscle torque is greater than that of the invertor muscles, the decrease in this ratio is likely due to the greater decline in evertor muscle torque. Therefore, special attention should be given to the endurance capacity and strength of the evertor muscles during sports exercises to minimize the impact of fatigue and reduce the risk of lateral ankle sprains, which are among the most common injuries in handball players (12).

The large effect size ($\eta_p^2 > 0.14$) demonstrates that the simulated handball game fatigue protocol had a robust and clinically significant impact on knee and ankle musculature peak torques. This large effect size suggests that the simulated handball game fatigue protocol induced significant changes in muscle performance, as measured by peak torques. This is clinically relevant because peak torque is a key indicator of muscle strength and function, which are critical for athletic performance

and injury prevention in handball players. For coaches and sports scientists, the large effect size underscores the importance of incorporating fatigue protocols into training regimens to better prepare athletes for the physical demands of competition. By replicating game-like fatigue conditions, athletes can develop greater resilience and adaptability in their knee and ankle musculature.

Despite the promising findings of this study, some limitations should be acknowledged. First, we limited the measurements to concentric contrations to calculate conventional ratios. However, a previous study have shown that the behavior of eccentric torque is similar to that of concentric in terms of conventional ratios (59). Second, we were unable to differentiate between central and peripheral causes of fatigue. Future studies should consider incorporating methods to identify the causes of fatigue within this type of protocol. Lastly, the conditions of handball match differ from those created in the simulation protocol used in this study. In handball, many injuries and injury-related situations result from collisions between players, which were not accounted for in the current study. However, this study focused on examining intrinsic factors within individuals identifying intrinsic mechanisms.

Conclusion

In general, the results of present study demonstrated that a handball match simulation-induced fatigue led to a decrease in the isokinetic peak torque of the hamstring, quadriceps, invertor, and evertor muscles. Furthermore, the findings revealed that fatigue reduced both the H:Q peak torque ratio and the E:I peak torque ratio. These changes could contribute to increased risk of ACL tears and lateral ankle sprains. Considering that hamstring muscles were more affected in the reduction of the H:Q peak torque ratio, it is recommended to focus on strengthening and improving the endurance of this muscle in handball exercises. Additionally, due to

the greater degrees of decrease in evertor muscles peak torque associated with the decrease in the E:I peak torque ratio, handball players are advised to prioritize enhancing the endurance and strength of the evertor muscles during their training sessions. Given that most lower limb injuries in handball occur in the final minutes of a match, it is recommended to specifically focus on training of hamstring and evertor muscles to increase their force production and endurance capacity to reduce injury risk and mitigate fatigue-related performance declines.

Acknowledgments

The authors thank all subjects who volunteered to participate in the present study.

Funding

This research did not receive any specific grant from funding agencies in the public, commercial, or not for profit sectors.

References

1. Póvoas SCA, Seabra AFT, Ascensão AAMR, Magalhães J, Soares JMC, Rebelo ANC. Physical and physiological demands of elite team handball. J Strength Cond Res. 2012;26(12):3365–75.
2. Michalsik LB, Aagaard P. Physical demands in elite team handball: Comparisons between male and female players. J Sports Med Phys Fitness. 2015;55(9):878–91.
3. Engebretsen L, Soligard T, Steffen K, Alonso JM, Aubry M, Budgett R, et al. Sports injuries and illnesses during the London Summer Olympic Games 2012. Br J Sports Med. 2013;47(7):407–14.
4. Michalsik LB. On-court physical demands and physiological aspects in elite team handball. Handball sports medicine: Basic science, injury management and return to sport. 2018;15–33.
5. Ament W, Verkerke GJ. Exercise and fatigue. Sports med. 2009;39:389–422.

6. Gandevia SC, Allen GM, Butler JE, Taylor JL. Supraspinal factors in human muscle fatigue: evidence for suboptimal output from the motor cortex. J Physiol. 1996;490(2):529–36.

7. Westerblad H, Lee JA, Lannergren J, Allen DG. Cellular mechanisms of fatigue in skeletal muscle. Am J Physiol Cell Physiol. 1991;261(2):C195–209.

8. Thorlund JB, Michalsik LB, Madsen K, Aagaard P. Acute fatigue-induced changes in muscle mechanical properties and neuromuscular activity in elite handball players following a handball match. Scand J Med Sci Sports. 2008;18(4):462–72.

9. Póvoas SCA, Ascensão AAMR, Magalhães J, Seabra AFT, Krustrup P, Soares JMC, et al. Analysis of fatigue development during elite male handball matches. J Strength Cond Res. 2014;28(9):2640–8.

10. Bedo BLS, Pereira DR, Moraes R, Kalva-Filho CA, Will-de-Lemos T, Santiago PRP. The rapid recovery of vertical force propulsion production and postural sway after a specific fatigue protocol in female handball athletes. Gait Posture. 2020;77:52–8.

11. Thorlund JB, Michalsik LB, Madsen K, Aagaard P. Acute fatigue-induced changes in muscle mechanical properties and neuromuscular activity in elite handball players following a handball match. Scand J Med Sci Sports. 2008;18(4):462–72.

12. Langevoort G, Myklebust G, Dvorak J, Junge A. Handball injuries during major international tournaments. Scand J Med Sci Sports. 2007;17(4):400–7.

13. Martín-Guzón I, Muñoz A, Lorenzo-Calvo J, Muriarte D, Marquina M, De la Rubia A. Injury prevalence of the lower limbs in handball players: A systematic review. Int J Environ Res Public Health. 2021;19(1):332.

14. Edouard P, Chatard JC, Fourchet F, Collado H, Degache F, Leclair A, et al. Invertor and evertor strength in track and field athletes with functional ankle instability. Isokinet Exerc Sci. 2011;19(2):91–6.

15. Ersoz M, Atalay NS, Kumbara F, Akyuz M. Investigation of effect of age, gender and limb dominance on ankle evertor/invertor peak torque ratios of healthy volunteers. J Phys Ther Sci. 2009;21(3):263–7.

16. Aagaard P, Simonsen EB, Trolle M, Bangsbo J, Klausen K. Isokinetic hamstring/quadriceps strength ratio: influence from joint angular velocity, gravity correction and contraction mode. Acta Physiol Scand. 1995;154(4):421–7.

17. Sangnier S, Tourny-Chollet C. Comparison of the decrease in strength between hamstrings and quadriceps during isokinetic fatigue testing in semiprofessional soccer players. Int J Sports Med. 2007;28(11):952–7.

18. van Melick N, van der Weegen W, van der Horst N. Quadriceps and hamstrings strength reference values for athletes with and without anterior cruciate ligament reconstruction who play popular pivoting sports, including soccer, basketball, and handball: a scoping review. J Orthop Sports Phys Ther. 2022;52(3):142–55.

19. Pontaga I. Ankle joint evertor–invertor muscle torque ratio decrease due to recurrent lateral ligament sprains. Clin Biomech. 2004;19(7):760–2.

20. Cozette M, Leprêtre PM, Doyle C, Weissland T. Isokinetic Strength Ratios: Conventional Methods, Current Limits and Perspectives. Front Physiol. 2019;10.

21. Gleeson NP, Mercer TH. The utility of isokinetic dynamometry in the assessment of human muscle function. Sports med. 1996;21:18–34.

22. Faul F, Erdfelder E, Lang AG, Buchner A. G*Power 3: a flexible statistical power analysis program for the social, behavioral, and biomedical sciences. Behav Res Methods. 2007 May;39(2):175–91.

23. Andrade MS, de Carvalho Koffes F, Benedito-Silva AA, da Silva AC, de Lira CAB. Effect of fatigue caused by a simulated handball game on ball throwing velocity, shoulder muscle strength and balance ratio: a prospective study. BMC Sports Sci Med Rehabil. 2016;8:1–7.

24. Esmaeili H, Sharifi A. Bilateral asymmetry of hamstring to quadriceps isokinetic torque ratio in different angular velocities in professional basketball players. Knee. 2022 Aug 1;37:3–9.

25. Aydoğ E, Aydoğ ST, Çakci A, Doral MN. Reliability of isokinetic ankle inversion-and eversion-strength measurement in neutral foot position, using the Biodex dynamometer. Knee Surg Sports Traumatol Arthrosc. 2004;12:478–81.

26. Fong DTP, Ha SCW, Mok KM, Chan CWL, Chan KM. Kinematics analysis of ankle inversion ligamentous sprain injuries in sports: five cases from televised tennis competitions. Am J Sports Med. 2012;40(11):2627–32.

27. Ameer MA. Kinematic comparison between single and double-leg jump landings in sagittal plane for male hand ball players. Turk J Sport Exer. 2016;18(3):108–15.

28. Borg G. Borg's perceived exertion and pain scales. Hum kinet; 1998.

29. Bove AM, Lynch AD, DePaul SM, Terhorst L, Irrgang JJ, Fitzgerald GK. Test-retest reliability of rating of perceived exertion and agreement with 1-repetition maximum in adults. J Orthop Sports Phys Ther. 2016;46(9):768–74.

30. Scott TJ, Black CR, Quinn J, Coutts AJ. Validity and reliability of the session-RPE method for quantifying training in Australian football: a comparison of the CR10 and CR100 scales. J Strength Cond Res. 2013;27(1):270–6.

31. Cohen J. Statistical power analysis for the behavioral sciences. routledge; 2013.

32. Pinto MD, Blazevich AJ, Andersen LL, Mil-Homens P, Pinto RS. Hamstring-to-quadriceps fatigue ratio offers new and different muscle function information than the conventional non-fatigued ratio. Scand J Med Sci Sports. 2018;28(1):282–93.

33. Massamba A, Hucteau E, Mallard J, Ducrocq GP, Favret F, Hureau TJ. Exercise-Induced Fatigue in Hamstring versus Quadriceps Muscles and Consequences on the Torque–Duration Relationship in Men. Med Sci Sports Exerc. 2022;54(12):2099–108.

34. Greco CC, Da Silva WL, Camarda SRA, Denadai BS. Fatigue and rapid hamstring/quadriceps force capacity in professional soccer players. Clin Physiol Funct Imaging. 2013;33(1):18–23.

35. Greig M, Siegler JC. Soccer-specific fatigue and eccentric hamstrings muscle strength. J Athl Train. 2009;44(2):180–4.

36. Delextrat A, Baker J, Cohen DD, Clarke N. Effect of a simulated soccer match on the functional hamstrings-to-quadriceps ratio in amateur female players. Scand J Med Sci Sports. 2013;23(4):478–86.

37. Greig M. The influence of soccer-specific fatigue on peak isokinetic torque production of the knee flexors and extensors. Am J Sports Med. 2008;36(7):1403–9.

38. Behrens M, Mau-Moeller A, Wassermann F, Bruhn S. Effect of fatigue on hamstring reflex responses and posterior-anterior tibial translation in men and women. PLoS One. 2013;8(2):e56988.

39. Nocella M, Colombini B, Benelli G, Cecchi G, Bagni MA, Bruton J. Force decline during fatigue is due to both a decrease in the force per individual cross-bridge and the number of cross-bridges. J Physiol. 2011;589(13):3371–81.

40. Gandevia SC. Spinal and supraspinal factors in human muscle fatigue. Physiol Rev. 2001;81(4):1725–89.

41. Fitts RH. Cellular mechanisms of muscle fatigue. Physiol Rev. 1994;74(1):49–94.

42. de Oliveira Pires F. Thomas Kuhn's 'Structure of Scientific Revolutions' applied to exercise science paradigm shifts: example including the Central Governor Model. Br J Sports Med. 2013;47(11):721–2.

43. Noakes TD. Physiological models to understand exercise fatigue and the adaptations that predict or enhance athletic performance. Scand J Med Sci Sports. 2000;10(3):123–45.

44. Marshall PWM, Lovell R, Knox MF, Brennan SL, Siegler JC. Hamstring fatigue and muscle activation changes during six sets of Nordic hamstring exercise in amateur soccer players. J Strength Cond Res. 2015;29(11):3124–33.

45. Bachasson D, Decorte N, Wuyam B, Millet GY, Verges S. Central and peripheral quadriceps fatigue in young and middle-aged untrained and endurance-trained men: A comparative study. Exp Biol Med. 2016;241(16):1844–52.

46. Pinto MD, Blazevich AJ, Andersen LL, Mil-Homens P, Pinto RS. Hamstring-to-quadriceps fatigue ratio offers new and different muscle function information than the conventional non-fatigued ratio. Scand J Med Sci Sports. 2018;28(1):282–93.

47. Chen TC, Lin KY, Chen HL, Lin MJ, Nosaka K. Comparison in eccentric exercise-induced muscle damage among four limb muscles. Eur J Appl Physiol. 2011;111:211–23.

48. Hamada T, Sale DG, MacDougall JD, Tarnopolsky MA. Interaction of fibre type, potentiation and fatigue in human knee extensor muscles. Acta Physiol Scand. 2003;178(2):165–73.

49. Smeets A, Malfait B, Dingenen B, Robinson MA, Vanrenterghem J, Peers K, et al. Is knee neuromuscular activity related to anterior cruciate ligament injury risk? A pilot study. Knee. 2019;26(1):40–51.

50. Akaras E, Güzel NA, Çobanoğlu G, Ecemiş ZB, Şimşek BÜ. The effect of hamstring/quadriceps muscle strength ratio on plantar pressure distribution-A pilot study. Turk J of Kin. 2024;10(4):298–305.

51. Nagel A, Fernholz F, Kibele C, Rosenbaum D. Long distance running increases plantar pressures beneath the metatarsal heads: A barefoot walking investigation of 200 marathon runners. Gait Posture. 2008;27(1):152–5.

52. Sangnier S, Tourny-Chollet C. Comparison of the decrease in strength between hamstrings and quadriceps during isokinetic fatigue testing in semiprofessional soccer players. Int J Sports Med. 2007;28(11):952–7.

53. Lin YH, Li CW, Tsai LY, Liing RJ. The effects of muscle fatigue and proprioceptive deficits on the passive joint senses of ankle inversion and eversion. Isokinet Exerc Sci. 2008;16(2):101–5.

54. Rodrigues KA, Soares RJ, Tomazini JE. The influence of fatigue in evertor muscles during lateral ankle sprain. The Foot. 2019;40:98–104.

55. Silva BARS, Martinez FG, Pacheco AM, Pacheco I. Effects of the exercise-induced muscular fatigue on the time of muscular reaction of the fibularis in healthy individuals. Rev Bras Med Esporte. 2006;12:85–9.

56. Sandrey MA, Kent TE. The effects of eversion fatigue on frontal plane joint position sense in the ankle. J Sport Rehabil. 2008;17(3):257–68.

57. Castillo GB, Brech GC, Luna NMS, Tarallo FB, Soares-Junior JM, Baracat EC, et al. Influence of invertor and evertor muscle fatigue on functional jump tests and postural control: A prospective cross-sectional study. Clinics. 2022;77:100011.

58. Kaminski TW, Hartsell HD. Factors contributing to chronic ankle instability: a strength perspective. J Athl Train. 2002;37(4):394.

59. Calmels PM, Nellen M, van der Borne I, Jourdin P, Minaire P. Concentric and eccentric isokinetic assessment of flexorextensor torque ratios at the hip, knee, and ankle in a sample population of healthy subjects. Arch Phys Med Rehabil. 1997;78(11):1224–30.

Dear Editor,

Thank you very much for the opportunity to submit the revised version of the manuscript, "**Effect of simulated handball match-induced fatigue on isokinetic hamstring-to-quadriceps ratio and invertor-to-evertor ratio in professional players**" for consideration in the **PLOS One**. We deeply appreciate the time and effort you and the reviewers have dedicated to evaluating our work and providing valuable feedback. We have carefully addressed each comment and made the necessary revisions to improve the quality of the manuscript. We hope that the revised version meets the high standards of the journal. Please find our detailed responses to each reviewer's comment below. Revised sections are marked and highlighted for clarity.

Reviewer #1:

The purpose of this quasi-experimental study was to examine the effects of a functional fatigue protocol in handball on muscle group torque for the knee extensors and flexors and ankle invertors and evertors. There appeared to be a need for this study and the study purpose was clear. More information is needed in the methods section. There is some confusion in the presentation of the results, although figures and tables complement the narrative. More specific details are needed in the discussion and a limitations section is needed as well. A more developed presentation on central and peripheral fatigue is needed in the introduction which will serve a section of the discussion well. There were several word choice and word tense errors, as well as errors and inconsistencies in the reference section. See pdf for details comments and questions.

Thank you very much for your thorough evaluation and thoughtful suggestions. We greatly value your feedback, and we believe that the revisions we have made in response to your comments, especially in the methodology section, have significantly strengthened our manuscript. Below, we highlight the key changes implemented in response to your comments:

- Regarding intermittent sport:

Intermittent sport, often referred to as intermittent exercise or interval training, involves alternating periods of high-intensity activity with periods of lower-intensity activity or rest. So, Handball is an intermittent sport.

- Your introduction to the concept of fatigue is superficial and appears to only apply to peripheral fatigue. Please more fully develop the concepts of peripheral and central fatigue first, then perhaps show the readers that the fatigue you are interested in mostly is related to peripheral fatigue. This needs to be well-referenced.

We have now expanded the introduction and discussion sections to include both peripheral and central fatigue.
We have also added the following to the manuscript:

- Sample size calculation
- Inclusion criteria
- General warm up
- A reference on the test-retest reliability of Borg scale measure
- Peak torque per body mass considered for measurements
- A brief description of the problem and the rationale/need for this study before restating the purpose
- Expanded operational definitions of general and local fatigue in the discussion and introduction
- Limitation section

Reviewer #2:

Thank you very much for your detailed review and constructive feedback. We appreciate your insights, which have helped strengthen our manuscript. The comments have been addressed and highlighted in the text. Please see our responses to each comment below.

1) The authors should justify why eccentric torque was not measured or acknowledge it as a limitation.

We have acknowledged this as a limitation in the manuscript.

2) Consider adding physiological fatigue markers (if available) to validate fatigue protocol

We have added physiological markers (Table 1) and expanded the discussion by adding the following:

"The proposed fatigue protocol was designed to induce high levels of physiological and psychological stress, ultimately leading to exhaustion. This was evident from the heart rate (HR) at exhaustion, which reached 186.31 ± 7.4 bpm, approximately 96% of the predicted maximum HR. Additionally, participants reported maximum values on the RPE score of 20, further confirming the extreme cardiovascular stress induced by the protocol. These findings, along with the observed reductions in quadriceps, hamstring invertor and evertor muscles peak torque per body mass and their respective ratios, strongly suggest that the protocol effectively induces fatigue. These results indicate that this protocol reliably simulates fatigue in handball players, making it a valuable tool for both training and research."

3) Discuss whether the reduction in H:Q and E:I ratios reaches clinically significant thresholds

We have added the following to the discussion section:

“The large effect size ($\eta_p^2 > 0.14$) demonstrates that the simulated handball game fatigue protocol had a robust and clinically significant impact on knee and ankle musculature peak torques. This large effect size suggests that the simulated handball game fatigue protocol induced significant changes in muscle performance, as measured by peak torques. This is clinically relevant because peak torque is a key indicator of muscle strength and function, which are critical for athletic performance and injury prevention in handball players. For coaches and sports scientists, the large effect size underscores the importance of incorporating fatigue protocols into training regimens to better prepare athletes for the physical demands of competition. By replicating game-like fatigue conditions, athletes can develop greater resilience and adaptability in their knee and ankle musculature.”

4) if possible, provide effect size interpretations and confidence intervals

They have been added to the tables as suggested.

5) Ensure consistency in statistical reporting (rounding, decimal places, p-values)

We have reviewed the statistical reporting for consistency.

6) if possible, consider adding a sample size power analysis

It was added as suggested

7) Expand discussion with comparisons to other team sports

We have included compressions with soccer where it was relevant.

8) Provide practical training recommendations for injury prevention

We have implemented these recommendations in the discussion.

9) You can benefit from the article https://doi.org/10.31459/turkjkin.1576269. in discussion section: When interpreting H:Q ratio reductions and their biomechanical consequences. or in introduction Section: To support the importance of the H:Q ratio in lower limb function.

Thank you so much for providing this valuable reference. We used this reference in the discussion section as suggested:

“Additionally, it has been shown that a decreased H:Q is associated with increased loading on the forefoot and toe regions (50). The increased loading could lead to increased risk of stress fracture in the forefoot region (51).”
