# Supplementary material for: Effect of simulated handball match-induced fatigue on isokinetic hamstring-to-quadriceps ratio and evertor-to-invertor ratio in professional players
Source: PLoS One. 2025 May 19;20(5):e0323978. doi: 10.1371/journal.pone.0323978 (PMC12088022; doi:10.1371/journal.pone.0323978)
Supplement: Attachment — Submitted filename: Response letter (2).docx [file pone.0323978.s005.docx]

Dear Dr. Mousavi,

Thank you again for considering our manuscript for publication in PLOS ONE and for giving us the opportunity to revise and improve the quality of our manuscript. We truly appreciate the thoughtful feedback provided by both reviewers. In response, we have carefully revised the manuscript to address Reviewer #1's comments, and all changes have been clearly highlighted in the text. We believe these revisions significantly enhance the clarity of the manuscript, and we hope it now meets the high standards of PLOS ONE.

We look forward to receiving your feedback and greatly appreciate the time and effort you've dedicated to our manuscript.

Sincerely,
Dr. Hamed Esmaeili

----------------

Reviewer #1: Thank you for addressing the many issues identified. Additional word choice, word tense, and spelling errors remain, some of which were in your highlighted sections so look carefully for my comments. The biggest error was found in the introduction in an incorrect definition of central and peripheral fatigue (see comment and a suggested reference). What was odd was that you correctly discussed central and peripheral fatigue in the discussion section. See pdf for specific comments.

Dear reviewer,
Thank you again for reviewing our manuscript and providing your invaluable feedback. We have carefully revised the manuscript to address your suggestions and have clarified the definitions of central and peripheral fatigue in the introduction, incorporating the excellent reference you recommended. Your insights have been incredibly helpful, and we believe these changes have greatly improved the manuscript.
