# Supplementary material for: Effect of simulated handball match-induced fatigue on isokinetic hamstring-to-quadriceps ratio and evertor-to-invertor ratio in professional players
Source: PLoS One. 2025 May 19;20(5):e0323978. doi: 10.1371/journal.pone.0323978 (PMC12088022; doi:10.1371/journal.pone.0323978)
Supplement: Attachment — Submitted filename: Pone Reviewer Comments-bb.docx [file pone.0323978.s004.docx]

**Dear Editor,**

Thank you very much for the opportunity to submit the revised version of the manuscript, “**Effect of simulated handball match-induced fatigue on isokinetic hamstring-to-quadriceps ratio and invertor-to-evertor ratio in professional players**” for consideration in the **PLOS One**. We deeply appreciate the time and effort you and the reviewers have dedicated to evaluating our work and providing valuable feedback. We have carefully addressed each comment and made the necessary revisions to improve the quality of the manuscript. We hope that the revised version meets the high standards of the journal. Please find our detailed responses to each reviewer’s comment below. Revised sections are marked and highlighted for clarity.

---------------------

Reviewer #1:

The purpose of this quasi-experimental study was to examine the effects of a functional fatigue protocol in handball on muscle group torque for the knee extensors and flexors and ankle invertors and evertors. There appeared to be a need for this study and the study purpose was clear. More information is needed in the methods section. There is some confusion in the presentation of the results, although figures and tables complement the narrative. More specific details are needed in the discussion and a limitations section is needed as well. A more developed presentation on central and peripheral fatigue is needed in the introduction which will serve a section of the discussion well. There were several word choice and word tense errors, as well as errors and inconsistencies in the reference section. See pdf for details comments and questions.

Thank you very much for your thorough evaluation and thoughtful suggestions. We greatly value your feedback, and we believe that the revisions we have made in response to your comments, especially in the methodology section, have significantly strengthened our manuscript. Below, we highlight the key changes implemented in response to your comments:

- Regarding intermittent sport:

Intermittent sport, often referred to as intermittent exercise or interval training, involves alternating periods of high-intensity activity with periods of lower-intensity activity or rest. So, Handball is an intermittent sport.

- Your introduction to the concept of fatigue is superficial and appears to only apply to peripheral fatigue. Please more fully develop the concepts of peripheral and central fatigue first, then perhaps show the readers that the fatigue you are interested in mostly is related to peripheral fatigue. This needs to be well-referenced.

We have now expanded the introduction and discussion sections to include both peripheral and central fatigue.
We have also added the following to the manuscript:

- Sample size calculation
- Inclusion criteria
- General warm up
- A reference on the test-retest reliability of Borg scale measure
- Peak torque per body mass considered for measurements
- A brief description of the problem and the rationale/need for this study before restating the purpose
- Expanded operational definitions of general and local fatigue in the discussion and introduction
- Limitation section

---------------------

Reviewer #2:

Thank you very much for your detailed review and constructive feedback. We appreciate your insights, which have helped strengthen our manuscript. The comments have been addressed and highlighted in the text. Please see our responses to each comment below.

1) The authors should justify why eccentric torque was not measured or acknowledge it as a limitation.

We have acknowledged this as a limitation in the manuscript.

2) Consider adding physiological fatigue markers (if available) to validate fatigue protocol

We have added physiological markers (Table 1) and expanded the discussion by adding the following:

“The proposed fatigue protocol was designed to induce high levels of physiological and psychological stress, ultimately leading to exhaustion. This was evident from the heart rate (HR) at exhaustion, which reached 186.31 ± 7.4 bpm, approximately 96% of the predicted maximum HR. Additionally, participants reported maximum values on the RPE score of 20, further confirming the extreme cardiovascular stress induced by the protocol. These findings, along with the observed reductions in quadriceps, hamstring invertor and evertor muscles peak torque per body mass and their respective ratios, strongly suggest that the protocol effectively induces fatigue. These results indicate that this protocol reliably simulates fatigue in handball players, making it a valuable tool for both training and research.”

3) Discuss whether the reduction in H:Q and E:I ratios reaches clinically significant thresholds

We have added the following to the discussion section:

“The large effect size ($\eta_{p}^{2}$ > 0.14) demonstrates that the simulated handball game fatigue protocol had a robust and clinically significant impact on knee and ankle musculature peak torques. This large effect size suggests that the simulated handball game fatigue protocol induced significant changes in muscle performance, as measured by peak torques. This is clinically relevant because peak torque is a key indicator of muscle strength and function, which are critical for athletic performance and injury prevention in handball players. For coaches and sports scientists, the large effect size underscores the importance of incorporating fatigue protocols into training regimens to better prepare athletes for the physical demands of competition. By replicating game-like fatigue conditions, athletes can develop greater resilience and adaptability in their knee and ankle musculature.”

4) if possible, provide effect size interpretations and confidence intervals

They have been added to the tables as suggested.

5) Ensure consistency in statistical reporting (rounding, decimal places, p-values)

We have reviewed the statistical reporting for consistency.

6) if possible, consider adding a sample size power analysis

It was added as suggested

7) Expand discussion with comparisons to other team sports

We have included compressions with soccer where it was relevant.

8) Provide practical training recommendations for injury prevention

We have implemented these recommendations in the discussion.

9) You can benefit from the article https://doi.org/10.31459/turkjkin.1576269. in discussion section: When interpreting H:Q ratio reductions and their biomechanical consequences. or in introduction Section: To support the importance of the H:Q ratio in lower limb function.

Thank you so much for providing this valuable reference. We used this reference in the discussion section as suggested:

“Additionally, it has been shown that a decreased H:Q is associated with increased loading on the forefoot and toe regions (50). The increased loading could lead to increased risk of stress fracture in the forefoot region (51).”
